# The reaction of acetamiprid with OH radicals in the environment: a theoretical study†

**DOI:** 10.1039/d5ra02754c

**Published:** 2025-06-06

**Authors:** Quan V. Vo, Nguyen Thi Hoa, Nguyen Thanh Vinh, Adam Mechler

**Affiliations:** a The University of Danang – University of Technology and Education Danang 550000 Vietnam vvquan@ute.udn.vn; b Faculty of Pharmacy, University of Pécs Pécs H-7624 Hungary; c Department of Biochemistry and Chemistry, La Trobe University Victoria 3086 Australia

## Abstract

The chemical fate of acetamiprid (AMP), a neonicotinoid pesticide, is determined by photo-oxidation: a combination of radical degradation pathways driven by the action of hydroxyl radicals. This study utilizes quantum chemical calculations to investigate the reaction of AMP with hydroxyl radicals in atmospheric, lipidic, and aqueous media. It was shown that the degradation process has a steep temperature dependence with the overall rate constant decreasing from 9.04 × 10^9^ to 5.01 × 10^9^ M^−1^ s^−1^ in the temperature range of 253–323 K thus AMP lifetime in the gas phase varies from 17.26 to 41.37 hours. In lipid media, the AMP + HO˙ reaction exhibited an overall rate constant *k*_overall_ of 1.63 × 10^8^ M^−1^ s^−1^, while in water, it was 2.95 × 10^8^ M^−1^ s^−1^, closely matching the experimentally measured rate constant (*k*_exp_ = 7.59 × 10^8^ M^−1^ s^−1^). In natural water, where hydroxyl radical concentrations range from 10^−18^ to 10^−15^ M, AMP degradation is predicted to occur over approximately 6.47 × 10^2^ to 1.06 × 10^6^ hours at 273–373 K, corresponding to a range of ∼27 days to ∼121 years. Across all examined media and temperature conditions, the AMP + HO˙ reaction followed primarily the hydrogen transfer mechanism, with a minor role also played by the radical adduct formation pathway.

## Abstract 2

In the environment, acetamiprid undergoes moderate degradation *via* hydroxyl radicals, leading to the formation of H-abstraction products.

## Graphical abstract

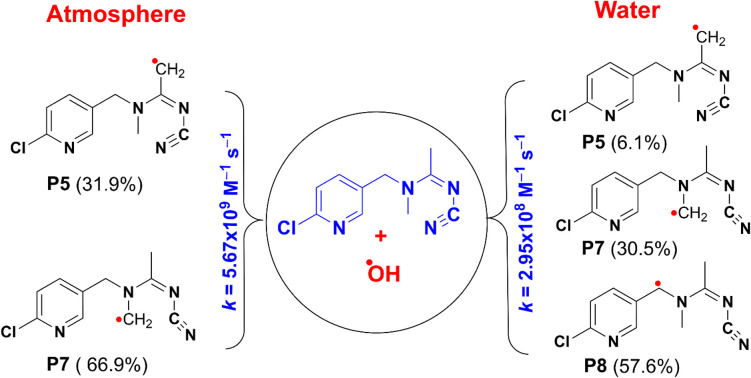

## 1 Introduction

**Fig. 1:**
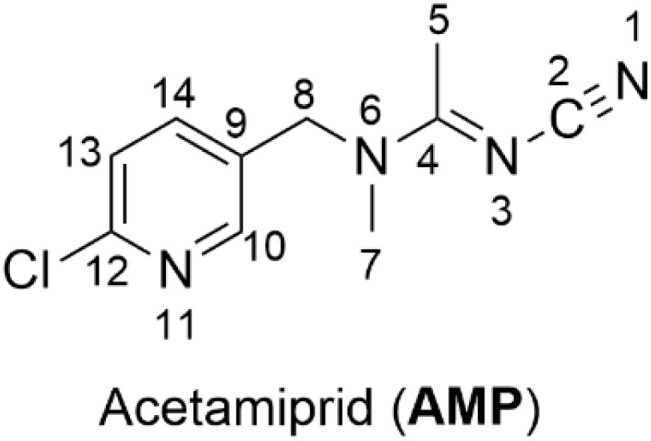
Structure of AMP.

Neonicotinoid insecticides are frequently used as seed coatings to reduce crop losses. These compounds are neurotoxins, attacking the central nervous system. Ultimately, neonicotinoids cause paralysis by acting as selective agonists that bond to acetylcholine receptors (AChRs) and disrupt neural function through overstimulation. They are widely used in pest control due to their high potency to disrupt the nervous systems of insects.^1,2^ (*E*)-*N*-(6-Chloro-3-pyridylmethyl)-*N*′-cyano-*N*-methylacetamidine, commonly referred to as acetamiprid (AMP), shown in Fig. 1, is a pesticide classified within the neonicotinoid insecticide group. It is among the most widely used insecticides in modern agricultural practices.^3–5^ Due to its widespread application, this micropollutant has been detected in surface water^6,7^ as well as wastewater^8,9^ samples globally. The presence of AMP in the environment and thus long-term exposure may pose potential risks to human health such as through DNA/RNA damage.^10^

Despite its low vapor pressure (∼1.7 × 10^−6^ Pa), acetamiprid exhibited the highest average atmospheric exposure concentration (0.05–3.0 μg m^−3^) among six commonly used pesticides, including acetamiprid, difenoconazole, thiazophos, isoprocarb, malathion, and pyridaben.^11^ In comparison to imidacloprid, acetamiprid demonstrated greater persistence in greenhouse air post-application, particularly under conditions of limited ventilation.^12^ Additionally, acetamiprid can be adsorbed and accumulated in airborne fine particulate matter.^13^ This particulate matter may serve as a significant carrier for the atmospheric transport of acetamiprid, thereby contributing to human exposure to airborne pesticides. Consequently, the prolonged persistence of acetamiprid in the greenhouse atmosphere may pose substantial long-term risks to the atmospheric systems and associated ecosystems; however, the degradation of AMP in the atmospheric environment has not been fully studied yet.

AMP persists at concentrations ranging from ng L^−1^ to μg L^−1^ across various organic waste matrices, including soils with high organic matter content,^14,15^ sewage sludge, and organic waste originating from wastewater treatment facilities.^16,17^ Furthermore, residual acetamiprid has been detected in agricultural organic waste, such as animal manure and plant residues.^4,15^ Despite its persistence, the degradation of AMP in such lipid-rich media remains underexplored, highlighting a critical knowledge gap that warrants further investigation.

Hydroxyl radical is a powerful oxidizing agent with high reactivity towards organic substrates. Hydroxyl radicals initiate the main natural breakdown pathways of organic molecules, even though their natural steady-state concentrations are modest, ranging from 10^−18^ to 10^−15^ M.^18,19^ Due to their potent oxidative properties, hydroxyl radicals are also used as primary oxidants in advanced oxidation processes for the destruction of neonicotinoid pesticides, including AMP, in industrial effluent.^20–25^

The HO˙ + AMP reaction rate constants in water were experimentally determined to fall in the range of 7.59 × 10^8^ to 2.1 × 10^9^ M^−1^ s^−1^.^17,20,26^ Previous studies concluded that the degradation of AMP by HO/O_2_ radicals can occur at the C7-H and C8-H bonds through a formal hydrogen transfer (FHT) reaction. This process results in the formation of (*E*)-*N*-(6-chloropyridin-3-yl(hydroxy)methyl)-*N*′-cyano-*N*-methylacetimidamide (*M*_W_ 238.7), (*E*)-*N*-((6-chloropyridin-3-yl)methyl)-*N*′-cyano-*N*-(hydroxymethyl)-acetimidamide (*M*_W_ 238.7),^17,26–28^ or (*E*)-6-chloro-*N*-(1-(cyanoimino)ethyl)-*N*-methylnicotinamide (*M*_W_ 236.7) and (*E*)-*N*-((6-chloropyridin-3-yl)methyl)-*N*′-cyano-*N*-formylacetimidamide (*M*_W_ 236.7).^27^ However, information regarding the formation of hydroxylated AMP (*M*_W_ 238.7) and the branching ratios of its intermediates remains limited.

As part of a series of studies on pesticide degradation using quantum chemical calculations,^29,30^ this work aims to assess the environmental persistence of AMP and examine the thermodynamic and kinetic properties of its hydroxyl radical-initiated degradation.

## 2 Computational methods

The Gaussian 16 software package was employed to perform the calculations for this study at the M06-2X/6-311++G(d,p) level of theory.^31^ This method is a proven way for precise calculations of thermodynamics and kinetics in current computational studies.^32–36^ The solvent effects of water were simulated using the SMD methodology,^32^ which is a common way of evaluating the radical scavenging properties of antioxidants. The methodology was benchmarked repeatedly against experimental results with a *k*_calc_/*k*_exp_ ratio of 0.3 to 2.9.^34,37–41^ It was recently suggested that a more realistic treatment of intramolecular rotation could improve the accuracy.^42^ However, due to the size and complexity of the system, the QM-ORSA level of accuracy was accepted as a necessary compromise.

Quantum Mechanics-based Overall Free Radical Scavenging Activity (QM-ORSA) method^41^ was used to perform the kinetic calculations.^34,43^ Under standard conditions of 1 M and varying ambient temperatures (253–323 K for the gas phase and 273–373 K for water), the rate constant (*k*) was determined using eqn (1) and transition state theory (TST) and details in Table S1, ESI.†^39,44–49^1
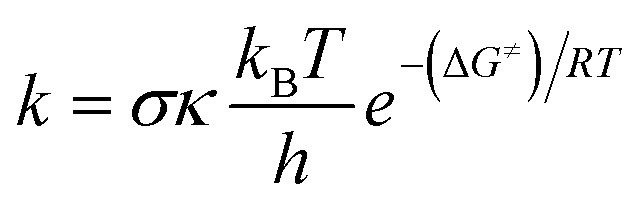


The Gibbs free energy of activation is denoted as Δ*G*^≠^, while *k*_B_ and *h* represent the Boltzmann constant and Planck constant, respectively. Tunneling corrections (*κ*), were computed using the Eckart barrier model.^50^*σ* stands for the reaction symmetry number.^51,52^

The radical adduct formation (RAF), or formal hydrogen transfer (FHT) pathways detailed in eqn (2) and (3) can provide the mechanistic underpinning of the reaction between AMP and HO˙, with consideration to the molecular structure:^29,34,39,43,53–55^

2 RAF: **AMP-H** + HO˙ → [**HO-AMP-H**]˙

3 FHT: **AMP-H** + HO˙ → **AMP**˙ + H_2_O

## 3 Results and discussion

### 3.1 Reaction of hydroxyl radical with AMP in the gas phase

#### 3.1.1 Thermodynamic evaluation

The molecular configuration of AMP indicates that the Me and CN substituents can undergo rotation about single bonds, resulting in multiple conformational isomers. Consequently, the five conformers with the lowest electronic energies were analyzed using the M06-2X/6-311++G(d,p) method, following an initial screening of potential AMP conformers (Fig. S1, ESI†).^56^ Among these, AMP exhibited the lowest Gibbs free energy, while conformers AMP-1 to AMP-4 displayed formation free energies exceeding that of AMP by 6.0–6.5 kcal mol^−1^. The relative populations of these conformers were determined through the Maxwell–Boltzmann distribution,^57,58^ confirming AMP as the predominant conformer (≈100%). Accordingly, this conformer was selected for further investigation.

The standard Gibbs free energy change (Δ*G*°) for each potential AMP + HO˙ reaction in the gas phase was calculated based on the FHT, and RAF mechanisms, with the results summarized in Table 1. The findings indicated that this reaction is generally spontaneous, except for the RAF mechanism at the N1, N3, N6, and N11 positions, where Δ*G*° values were positive. Electron-withdrawing substituents such as Cl and C≡N diminish the nitrogen atom's electron density, leading to instability of radical adducts at these positions. The higher energy cost of the addition reaction at N3/11 = C bonds is likely due to this effect. These results suggest that the AMP + HO˙ reaction in the gas phase can follow either FHT or RAF mechanisms (Δ*G*° < 0), highlighting the need for further kinetic analysis of these pathways.

**Table 1:** The calculated Δ*G*° (kcal mol^−1^) of the reaction between AMP with HO˙ radical following the FHT and RAF pathways at 298.15 K in the gas phase

Mechanisms	Positions	Δ*G*°
FHT	C5-H	−22.0
	C7-H	−25.3
	C8-H	−28.8
RAF	N1	25.0
	N3	30.5
	N6	1.7
	N11	21.5
	C2	−17.6
	C4	−8.1
	C9	−10.1
	C10	−14.1
	C12	−25.5
	C13	−10.0
	C14	−6.5

#### 3.1.2 Kinetic study

**Fig. 2:**
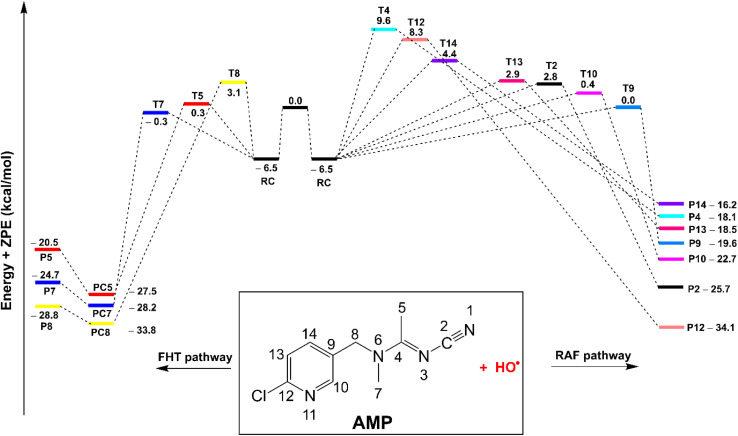
The potential energy surfaces of the AMP + HO˙ reaction at 0 K, in the gas phase (PC: post-complexes).

The outcomes of the kinetic analysis of the spontaneous reactions of AMP with HO˙ radicals in the gas phase are presented in Table 2, while the computed potential energy surfaces (PES) are depicted in Fig. 2. The chemical reaction proceeds *via* the formation of a pre-complex (RC) without an inherent reaction barrier, as shown in Fig. 2. The existence of the RC (RC-C5, Table S2, ESI†) was suggested by the observation that the relative energy of the transition state (T7) is lower than that of the reactants. Consequently, the most energetically favorable RC (RC-C5) served as the basis for evaluating the kinetics of the AMP + HO˙ reaction. The RAF process was observed to prefer the site of least sterically hindrance on the aromatic ring, leading to the most stable transition state compared to the alternative possibilities.

**Table 2:** Calculated Δ*G*^≠^ (kcal mol^−1^), rate constants (*k*_Eck_ and *k*_overall_ M^−1^ s^−1^), tunneling corrections (*κ*) and branching ratios (*Γ*, %) of the reaction between HO˙ and AMP reactions at 298.15 K

Mechanism		Δ*G*^≠^	*κ*	*k*_Eck_	*Γ*	Products
FHT	C5-H	6.9	10.4	1.81 × 10^9^	31.9	P5
	C7-H	5.4	1.8	3.79 × 10^9^	66.9	P7
	C8-H	9.3	1.8	3.61 × 10^6^	0.1	P8
RAF	C2	9.3	1.6	1.39 × 10^6^	0.0	P2
	C4	17.4	1.2	1.39	0.0	P4
	C9	7.3	1.2	3.61 × 10^7^	0.6	P9
	C10	7.5	1.3	2.47 × 10^7^	0.4	P10
	C12	15.1	1.5	7.83 × 10^1^	0.0	P12
	C13	9.6	1.3	7.83 × 10^5^	0.0	P13
	C14	11.2	1.4	5.06 × 10^4^	0.0	P14
*k*_overall_ (AMP + HO˙)				**5.67 × 10** ^**9**^		

The HO˙ radicals form intermediates P2-14 by abstracting hydrogen through the C–H bonds located at C5, C7, and C8, or alternatively through T2, T4, T9, T10, T11, T12, T13 and T14 within the RAF pathway. The FHT pathway has some variation in the reaction barrier ranging from 6.2 to 9.6 kcal mol^−1^. Conversely, the RAF barrier for reactions at positions T2, T4, T9, T10, T11, T12, T13, and T14 range from 6.5 to 16.1 kcal mol^−1^ towards the corresponding products P2, P4, and P9-14. The H-abstraction of C5 and C7 bonds exhibited the lowest observed reaction barriers at 6.2 and 6.8 kcal mol^−1^, respectively, whereas that of RAF was observed at the C9 position, which had a value of 6.5 kcal mol^−1^. Consequently, the abstraction of hydrogen of the C5 and C7 bonds is the dominant degradation of AMP against HO˙ radicals in the gas phase.

The findings summarized in Table 2 indicate that HO˙ and AMP engage in rapid gas-phase interactions, characterized by an overall rate constant of *k*_overall_ = 5.67 × 10^9^ M^−1^ s^−1^, in accordance with the H-abstraction mechanism. Under the studied media, the RAF pathway did not play a role in the degradation of AMP by HO˙ radicals. Despite the relatively low reaction barrier of 6.5 kcal mol^−1^ for the C9 position (Fig. 2), the RAF reaction at C9 accounted for only 0.6% of the *k*_overall_ value. For the formation of the principal intermediates (as detailed in Table 2), the highest rate constants were recorded for C5 and C7, with values of *k*_app_ = 1.81 × 10^9^ and 3.79 × 10^9^ M^−1^ s^−1^, respectively. It is noteworthy that the tunneling correction (*κ*) for the FHT (C5-H) mechanism (*κ* = 10.4) is approximately 5.8 times greater than that for the C7/8−H mechanisms (*κ* = 1.8). This difference is attributed to the lower vibrational frequency of C5 (*ν* = −1482.54 cm^−1^) compared to C7 (*ν* = −744.10 cm^−1^) and C8 (*ν* = −741.06 cm^−1^), as detailed in Table S2 of the ESI file.† This resulted in the formation of P5 at a yield of 31.9% and P7 at a yield of 66.9%.

#### 3.1.3 The effect of temperature on the decomposition of AMP and its lifetimes in the gas phase

**Fig. 3:**
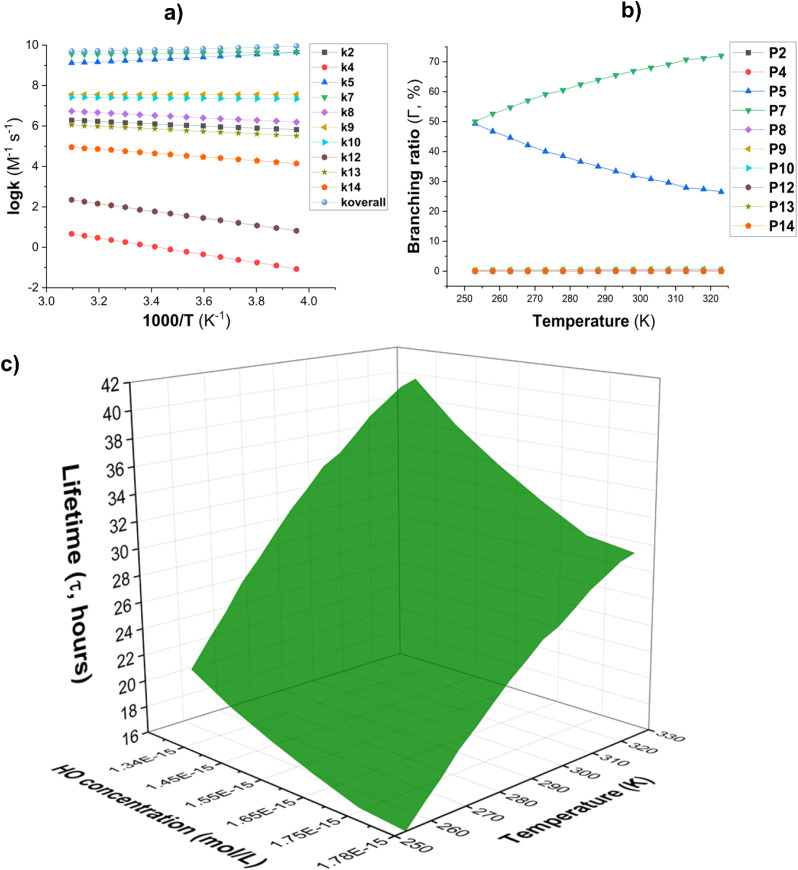
(a) The temperature influence on apparent rate constants (log *k*); (b) *Γ* values (%); (c) lifetime (*τ*, s) at 253–323 K.

To examine the effect of temperature on the reaction of AMP with HO˙ radicals in the gas phase, the kinetics of each mechanism were determined in the 253–323 K range. The results are depicted in Fig. 3. The rate constants of the FHT (C5 and C7) reaction decrease as the temperature in the gas phase increases (Fig. 3a) while the rate of RAF and FHT (C8) reactions increase. However, the *k*_overall_ value decreased (from 9.04 × 10^9^ to 5.01 × 10^9^ M^−1^ s^−1^) as the temperature rose from 253 to 323 K due to the dominance of the FHT reaction. The amount of intermediate P5 decreased from 49.3 to 26.5% as the temperature increased, as evidenced by the branching ratio (Fig. 3b). In parallel the fraction of intermediate P7 increased from 50.0 to 72.0%. The remaining products were omitted from Fig. 3b due to their negligible contribution to the overall rate constant. Thus, P5 and P7 remained the main intermediate products of the AMP + HO˙ reaction in the gas phase.

The lifetime (*τ*) of AMP was also investigated in the presence of HO˙ radicals at temperatures ranging from 253 to 323 K. The concentration of [HO˙] was [HO˙] = [1.34–1.78] × 10^−15^ M ([9.4 ± 1.3] × 10^5^ molecule per cm^3^, Fig. 3C).^59,60^ The findings indicate that AMP can easily decompose in the gaseous state within 17.26–41.37 hours in the temperature range of 253 to 323 K. The atmospheric decomposition of AMP becomes increasingly efficient as the ambient temperature increases.

### 3.2 Reaction of hydroxyl radical with AMP in lipid environment

The FHT and RAF mechanisms were used to directly calculate the kinetics of the AMP + HO˙ reaction in the lipidic medium (*i.e.*, pentyl ethanoate solvent) in all possible positions following the gas phase investigation. Table 3 shows the findings. It was determined that in the lipidic medium AMP can react with the hydroxyl radical at an overall rate constant of 1.63 × 10^8^ M^−1^ s^−1^. The FHT reaction is the predominant pathway with a *Γ* value exceeding 95.1%. The RAF reactions contributed only 1.2% to the overall rate constant. The HO˙ + AMP reaction was only slightly enhanced by the other FHT reaction of C8-H, with a *Γ* value of 3.6%. It was noted that the rate of HO˙ + AMP reaction in the lipid medium was comparable to the radical decomposition of the lipid itself (*k*_overall_ = 4.12 × 10^8^ M^−1^ s^−1^). Thus in lipid media AMP might not decompose. Even if neglecting this competition effect, the degradation rate of AMP in the lipid medium was approximately 34.8 times lower than that of the gas phase (Table 2). Thus, nonpolar media are not suitable environments for the degradation of AMP by HO˙ radicals.

**Table 3:** Calculated Δ*G*^≠^ (kcal mol^−1^), *κ*, *k*_app_, *k*_overall_ (M^−1^ s^−1^) and *Γ* (%) for the HO˙ + AMP reactions at 298.15 K in the lipid medium^a^^,^^b^

Mechanism		Δ*G*^≠^	*κ*	*k*_D_	*k*_app_	*Γ*	Products
FHT	C5-H	8.9	4.3	3.00 × 10^9^	2.50 × 10^7^	15.3	P5
	C7-H	7.4	1.8	3.10 × 10^9^	1.30 × 10^8^	79.8	P7
	C8-H	9.2	1.9	3.10 × 10^9^	5.90 × 10^6^	3.6	P8
RAF	C2	14.7	1.6	2.30 × 10^9^	1.70 × 10^2^	0.0	P2
	C4	18.2	1.3	2.20 × 10^9^	0.340	0.0	P4
	C9	9.0	1.2	2.40 × 10^9^	1.80 × 10^6^	1.1	P9
	C10	11.0	1.3	2.40 × 10^9^	7.00 × 10^4^	0.0	P10
	C12	15.1	1.5	2.30 × 10^9^	8.30 × 10^1^	0.0	P12
	C13	10.7	1.3	2.40 × 10^9^	1.20 × 10^5^	0.1	P13
	C14	12.4	1.4	2.30 × 10^9^	7.50 × 10^3^	0.0	P14
*k*_overall_ (AMP + HO˙)					**1.63 × 10** ^**8**^		

a *k*_overall_ = Σ*k*_app_.

b *Γ* = *k*_app_ × 100/*k*_overall_.

### 3.3 Reaction of hydroxyl radical with AMP in water

#### 3.3.1 The reaction of AMP with HO˙ in water

**Fig. 4:**
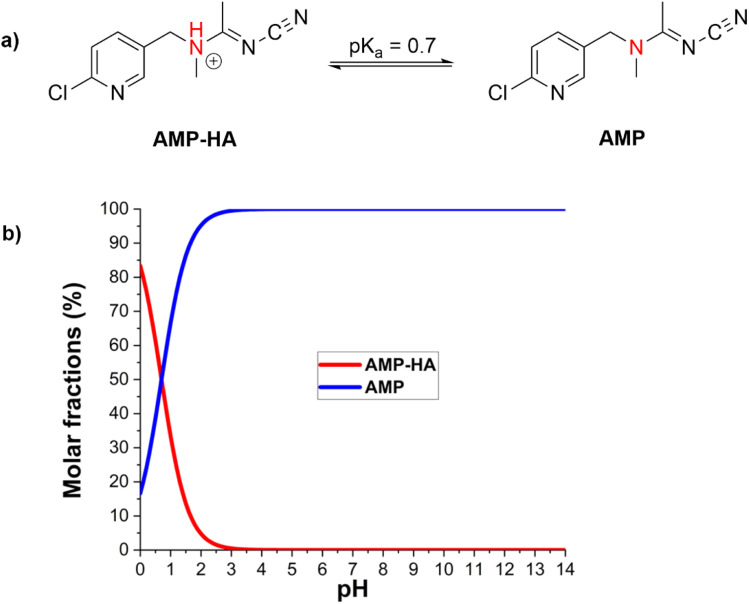
The acid dissociation equilibrium (a) and molar fractions (b) of AMP.

When organic molecules and free radical species interact in aqueous media, deprotonation is a critical factor.^29^ Consequently, it is imperative to take into account the deprotonation of AMP when assessing the effectiveness of water in the elimination of radicals. In the past, the p*K*_a_ value of AMP was documented as 0.7 (Fig. 4).^26,61,62^ Therefore, acetamiprid is in a neutral state (AMP, Fig. 4) in the natural aquatic environment (pH > 2). Consequently, the neutral state is the only one to consider when assessing the kinetics of the reaction of AMP with HO˙ radicals in an aqueous solution.

The interaction between AMP and HO˙ may proceed by RAF or FHT mechanisms. Thus, the kinetics of the AMP + HO˙ reaction in water were computed and are displayed in Table 4. The AMP + HO˙ reaction exhibited the *k*_overall_ of 2.95 × 10^8^ M^−1^ s^−1^. This aligns with the measured experimental rate constant (*k*_exp_ = 7.59 × 10^8^ M^−1^ s^−1^).^26^ The FHT mechanism dominated the reaction between AMP and HO˙ radicals, contributing 94.2% to the *k*_overall_. The primary contributors to the FHT reactions at the C8-H and C7-H positions exhibited *Γ* values of 57.6% and 30.5%, respectively. The two pathways together represented 88.1% of the total reactivity. The FHT reaction at C5-H contributed an additional 6.1% to the overall rate constant. The RAF mechanism contributed 5.8% to the overall rate constant, comprising 1.6% from the C9 position and 3.2% from the C10 position. Other RAF pathways (C2, C4, C12, C13, and C14) exhibited minimal contribution.

**Table 4:** Computed Δ*G*^≠^ (kcal mol^−1^), *κ*, *k*_app_, *k*_overall_ (M^−1^ s^−1^), and *Γ* (%) at 298.15 K, in the HO˙ + AMP in water^a^^,^^b^^,^^c^

Mechanism		Δ*G*^≠^	*κ*	*k*_D_	*k*_app_	*Γ*	
FHT	C5-H	9.2	5.2	2.90 × 10^9^	1.80 × 107	6.1	P5
	C7-H	7.8	2.5	3.00 × 10^9^	9.00 × 107	30.5	P7
	C8-H	7.1	2.5	2.90 × 10^9^	1.70 × 108	57.6	P8
RAF	C2	12.0	1.5	2.20 × 10^9^	1.40 × 10^4^	0.0	P2
	C4	16.3	1.3	2.10 × 10^9^	8.30	0.0	P4
	C9	8.4	1.1	2.30 × 10^9^	4.80 × 10^6^	1.6	P9
	C10	8.1	1.3	2.30 × 10^9^	9.50 × 10^6^	3.2	P10
	C12	12.7	1.3	2.30 × 10^9^	3.90 × 10^3^	0.0	P12
	C13	8.7	1.2	2.30 × 10^9^	3.00 × 10^6^	1.0	P13
	C14	11.2	1.4	2.20 × 10^9^	5.20 × 10^4^	0.0	P14
*k*_overall_ (AMP + HO˙)					**2.95 × 10** ^**8**^		

a *λ* (the nuclear reorganization energy, kcal mol^−1^).

b *k*_overall_ = Σ*k*_app_.

c *Γ* = *k*_app_ × 100/*k*_overall_.

The principal intermediates of the AMP + HO˙ reaction in the aqueous solution were P5 (6.1%), P7 (30.5%), and P8 (57.6%) as shown in Table 4, while those in the lipid medium (Table 3) were P5 (15.3%), P7 (79.8%) and P8 (3.6%).

#### 3.3.2 The effect of temperature on the decomposition of AMP and its lifetimes in water

**Fig. 5:**
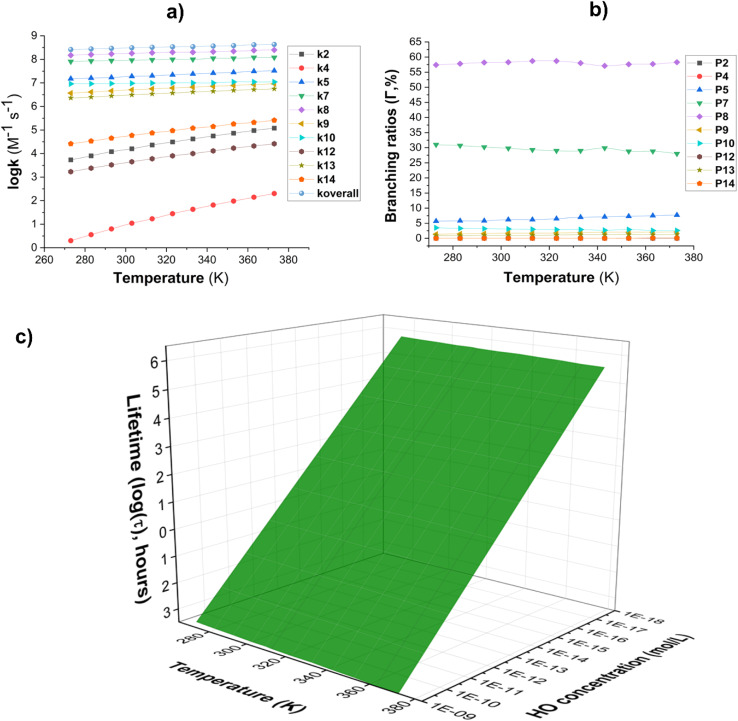
(a) Temperature-dependent apparent rate constants (log *k*); (b) branching ratio (*Γ*, %); (c) lifetime (log(*τ*), h, *τ* = 1/([HO] × *k*)) in water in the range of 273–373 K.

The rate constants for AMP breakdown by HO˙ radicals in water were calculated at temperatures ranging from 273 K to 373 K (Fig. 5a). The overall rate constant for the AMP + HO˙ reaction exhibited a slight reduction from 9.12 × 10^9^  M^−1^ s^−1^ at 273 K to 9.33 × 10^9^  M^−1^ s^−1^ at 373 K while demonstrating a stable temperature dependency. Concerning the branching ratio (Fig. 5b), it was observed that with rising temperature, the principal intermediates for the AMP + HO˙ reaction were P8 (57.4–58.3%), P7 (31.0–28.0%), and P5 (5.7–7.7%). As the temperature increased, the fraction of intermediate P8 remained stable, although the proportion of P7 had a modest decline and P5 saw an increase. That could be well explained with the formation of (*E*)-*N*-(6-chloropyridin-3-yl(hydroxy)methyl)-*N*′-cyano-*N*-methylacetimidamide (*M*_W_ 238.7), (*E*)-*N*-((6-chloropyridin-3-yl)methyl)-*N*′-cyano-*N*-(hydroxymethyl)-acetimidamide (*M*_W_ 238.7), or (*E*)-6-chloro-*N*-(1-(cyanoimino)ethyl)-*N*-methylnicotinamide (*M*_W_ 236.7) and (*E*)-*N*-((6-chloropyridin-3-yl)methyl)-*N*′-cyano-*N*-formylacetimidamide (*M*_W_ 236.7) in the experimental studies.^17,26–28^

Additional intermediates, namely P9 and P10, exhibited negligible contributions (1.4–2.2% and 2.6–3.5%, respectively) with small fluctuations throughout the studied temperature range. The results of the calculations indicated that the degradation of AMP by HO˙ radicals consistently generated the same primary intermediates (P8, P7, and P5) at all temperatures evaluated. Therefore, it is determined that the reaction mechanism remains substantially unchanged within the temperature range. The FHT pathways (represented by P7 and P8) are the primary mechanism of the reaction, while RAF pathways (such as P9 and P10) have a minor impact.

These findings emphasize the necessity of examining the AMP + HO˙ reaction mechanism in the aqueous phase in the context of multiple reaction pathways, such as FHT and RAF. Temperature-dependent data is essential for assessing the environmental fate of AMP under a variety of conditions.^63^

The lifetime (*τ*) of AMP in the presence of HO radicals in water at temperatures ranging from 273 to 383 K, with HO˙ concentrations of 10^−18^ to 10^−15^ M in natural water and 10^−10^ to 10^−9^ M in AOP-treated wastewater (Fig. 5c).^18,19^ It was found that the degradation of AMP in water takes place within a time frame of 6.47 × 10^−4^ to 1.06 × 10^6^ hours. In wastewater treated with AOP ([HO˙] = 10^−10^ to 10^−9^ M), the degradation of AMP is achieved rapidly, within 2.83 to 38.3 seconds. The rate of degradation increases with increasing temperature from 273 to 373 K. Consequently, the AOP approach is a highly effective method for the removal of AMP from aqueous systems. In aqueous aerosol particles, the HO˙ concentration is approximately 10^−11^ to 10^−13^ M.^64–66^ The lifetime of AMP in these particles ranges from 0.07 to 6.47 hours (at temperatures between 273 and 373 K). These results show that the ˙OH concentration is sufficient to drive significant oxidative processes in remote aerosols. The degradation of AMP in remote aerosols occurs at a significantly faster rate compared to that in the atmosphere, where the half-life ranges from 17.26 to 41.37 hours. Consequently, this appears to suggest that AMP is predominantly removed within aerosols rather than in the gas phase in the atmospheric environment.

In the natural aqueous solution ([HO˙] = 10^−18^ to 10^−15^ M), the degradation of AMP takes 6.47 × 10^2^ to 1.06 × 10^6^ hours (*i.e.* 0.07 hours to 121.43 years). For a given [HO˙], the lifetime of AMP declines as the temperature rises. As a result, the lifetime of AMP in the environment is estimated to be between 1.06 × 10^3^ to 1.06 × 10^6^ hours at a low temperature of 273 K. This amount may, however, reduce to 6.47 × 10^2^ to 6.47 × 10^5^ hours at 373 K. The data shows that *τ* shortens from 121.43 years at 273 K to 73.90 years at 373 K when [HO˙] = 10^−18^ M which confirms Arrhenius-based kinetic models that demonstrate temperature increases the rate of radical-driven deterioration.^67^ This trend is continuous across all HO˙ concentrations, revealing the major impact of temperature on the persistence of AMP in water.

## 4 Conclusion

The degradation of the AMP with HO˙ radicals under aqueous, lipid, and atmospheric conditions was investigated using DFT calculation. The results revealed that the reaction between hydroxyl radicals and AMP exhibited a decrease in the overall rate constant from 9.04 × 10^9^ to 5.01 × 10^9^ M^−1^ s^−1^ within the temperature range of 253–323 K. The degradation of AMP by hydroxyl radicals proceeded rapidly in the gas phase, with an estimated lifetime ranging from 17.26 to 41.37 hours. The AMP + HO˙ reaction in the lipid medium exhibited the *k*_overall_ of 1.63 × 10^8^ M^−1^ s^−1^, whereas that in water was 2.95 × 10^8^ M^−1^ s^−1^. This aligns with the measured experimental rate constant (*k*_exp_ = 7.59 × 10^8^ M^−1^ s^−1^). In natural water where the hydroxyl radical concentration ranges from 10^−18^ to 10^−15^ M, AMP degradation is expected to occur over a timescale of approximately 6.47 × 10^2^ to 1.06 × 10^6^ hours at 273–373 K, equivalent to 0.07 to 121.43 years. In all of the studied media and temperatures, the FHT reaction of C5, C7, and C8 defined the AMP + HO˙ reaction, whereas the RAF reaction contributed a minor role in the degradation, resulting in the main intermediates of P5, P7, and P8.

## Data availability

The data supporting this article have been included as part of the ESI.†

## Conflicts of interest

There are no conflicts to declare.

## Supplementary Material

RA-015-D5RA02754C-s001
