# Supplementary material for: The reaction of acetamiprid with OH radicals in the environment: a theoretical study
Source: RSC Adv. 2025 Jun 6;15(24):19236–44. doi: 10.1039/d5ra02754c (PMC12143200; doi:10.1039/d5ra02754c)
Supplement: RA-015-D5RA02754C-s001 [file RA-015-D5RA02754C-s001.pdf]

Supplementary Information (SI) for RSC Advances.
This journal is © The Royal Society of Chemistry 2025

Supporting Information (SI)

The reaction of acetamiprid with OH radicals in the environment: A theoretical study

Quan V. Vo[1*], Nguyen Thi Hoa[1], Nguyen Thanh Vinh,[2] and Adam Mechler[3]

[1]The University of Danang - University of Technology and Education, Danang 550000, Vietnam.
[2]Faculty of Pharmacy, University of Pécs, Pécs H-7624, Hungary
[3]Department of Biochemistry and Chemistry, La Trobe University, Victoria 3086, Australia.
*Corresponding authors: vvquan@ute.udn.vn;

Table of Contents

Table S1. The method to calculate rate constant following the conventional transition state theory S2

Figure S1. The typical conformers of AMP and the relative free energies ΔG° (in kcal/mol) compared to the AMP conformer S4

Table S2: The Cartesian coordinates, energies and imaginary frequency of AMP, RC and TS of the reaction between AMP with HO$^\bullet$ in the studied media (G: the gas phase; P: pentyl ethanoate; W: water) S5

References S25

Table S1. The method to calculate rate constant following the conventional transition state theory

The rate constant (k) was calculated by using the conventional transition state theory (TST) (at 298.15 K, 1M standard state) according to the equation (1):[1-5]

$$k = \sigma\kappa \frac{k_B T}{h} e^{-(\Delta G^{\neq})/RT} \quad (1)$$

Where: σ is the reaction symmetry number ,[6,7]

κ contains the tunneling corrections calculated using the Eckart barrier,[8]

k_B is the Boltzmann constant,

h is the Planck constant,

$\Delta G^{\neq}$ is the Gibbs free energy of activation.

The Marcus Theory was used to estimate the reaction barriers of SET reactions.[9-12] The free energy of reaction $\Delta G^{\neq}$ for the SET pathway was computed following the equations (2,3).

$$\Delta G^{\neq}_{SET} = \frac{\lambda}{4}\left(1 + \frac{\Delta G^{0}_{SET}}{\lambda}\right)^2 \quad (2)$$

$$\lambda \approx \Delta E_{SET} - \Delta G^{0}_{SET} \quad (3)$$

where ΔG_{SET} is the Gibbs energy of reaction, ΔE_{SET} is the non-adiabatic energy difference between reactants and vertical products for SET.[13,14]

For rate constants that were close to the diffusion limit a correction was applied to yield realistic results[15]. The apparent rate constants (k_{app}) were calculated following the Collins–Kimball theory in the solvents at 298.15K;[16] the steady-state Smoluchowski rate constant (k_D) for an irreversible bimolecular diffusion–controlled reaction was calculated following the literature as corroding to equations (4,5).[15,17]

$$k_{app} = \frac{k_{TST} k_D}{k_{TST} + k_D} \quad (4)$$

$$k_D = 4\pi R_{AB} D_{AB} N_A \quad (5)$$

where R_{AB} is the reaction distance, N_A is the Avogadro constant, and $D_{AB} = D_A + D_B$ (D_{AB} is the mutual diffusion coefficient of the reactants A and B),[16,18] where D_A or D_B is estimated using the

Stokes–Einstein formulation (6).[19,20]

$$D_{A\ or\ B} = \frac{k_{\mathrm{B}}T}{6\pi\eta a_{A\ or\ B}} \qquad (6)$$

η is the viscosity of the solvents (i.e. $\eta(H_2O) = 8.91\times10^{-4}$ Pa s, η(pentyl ethanoate) = 8.62×10^{-4} Pa s) and *a* is the radius of the solute.

The kinetic study requires different considerations. Water (dielectric constants, $\varepsilon = 78.35$) and pentyl ethanoate ($\varepsilon = 4.73$) are the *de facto* standard solvents in the literature to mimic the polar and nonpolar environments.[15,21-23] All transition states were characterized by the existence of only one single imaginary frequency. Intrinsic coordinate calculations (IRCs) were performed to ensure that each transition state is connected correctly with the pre-complex and post-complex.

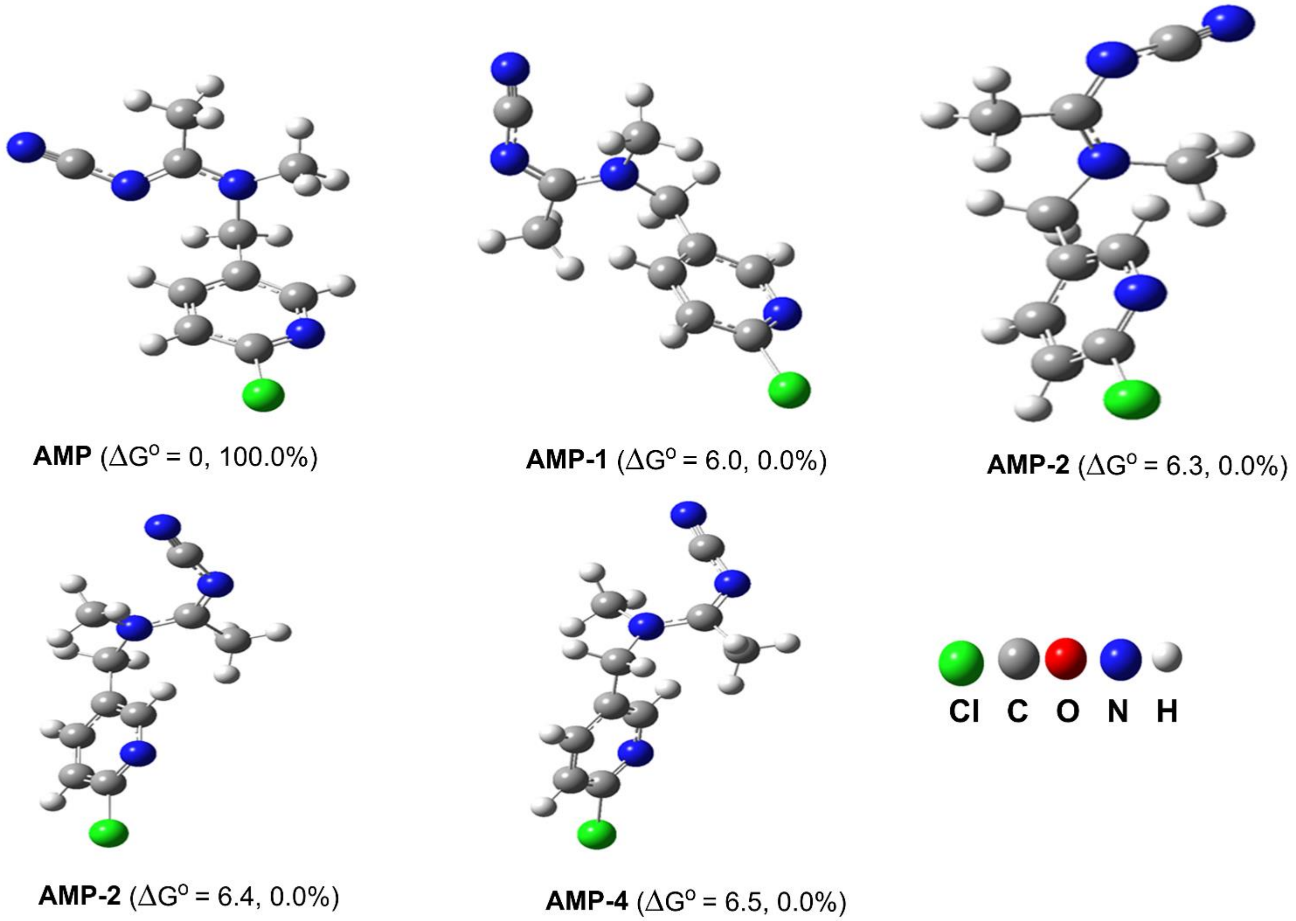

Figure S1. The typical conformers of AMP and the relative free energies ΔG° (in kcal/mol) compared to the AMP conformer

Table S2: The Cartesian coordinates, energies and imaginary frequency of AMP, RC and TS of the reaction between AMP with HO$^{\bullet}$ in the studied media (G: the gas phase; P: pentyl ethanoate; W: water)

**Name**	**AMP-G**
Cartesian Coordinates	Frequency and Energy
Cl -5.12883800 1.54695900 0.62260600<br>N 0.96685100 -0.91907100 -0.22803400<br>N -3.53048200 -0.34294200 -0.21218200<br>N 1.92881300 1.12528000 -0.52565700<br>N 3.59931300 2.87919700 0.02686700<br>C 0.05790500 -0.64003000 -1.34504300<br>C -1.23929000 -0.02464600 -0.87858200<br>C 1.85493400 0.02062500 0.16439300<br>C 0.60982900 -2.05410300 0.61159900<br>C -1.35197900 1.34405200 -0.61551100<br>C -2.36402000 -0.81174600 -0.66262800<br>C 2.70744300 -0.27466900 1.36939900<br>C -2.55424000 1.84827200 -0.15198000<br>C -3.60133800 0.94514800 0.03149100<br>C 2.83892500 2.03816600 -0.20246900<br>H -0.13881300 -1.58950100 -1.84768900<br>H 0.57245600 0.02187400 -2.03810100<br>H -0.17070000 -1.79101400 1.33345500<br>H 1.47719700 -2.43451600 1.14494300<br>H 0.23271600 -2.85203800 -0.02854800<br>H -0.50086600 1.99588500 -0.77694800<br>H -2.33459200 -1.87922900 -0.86810500<br>H 3.37804900 0.55950100 1.56337200<br>H 3.30297300 -1.17457000 1.20475500<br>H 2.08022200 -0.43632600 2.24820600<br>H -2.69246800 2.89943800 0.06039700	Zero-point correction= 0.203661 (Hartree/Particle)<br>Thermal correction to Energy= 0.218079<br>Thermal correction to Enthalpy= 0.219024<br>Thermal correction to Gibbs Free Energy= 0.160012<br>Sum of electronic and zero-point Energies= -1066.578294<br>Sum of electronic and thermal Energies= -1066.563876<br>Sum of electronic and thermal Enthalpies= -1066.562932<br>Sum of electronic and thermal Free Energies= -1066.621943
**Name**	**AMP-P**
Cartesian Coordinates	Frequency and Energy
Cl -5.27367900 1.42714500 0.46153400<br>N 0.94117300 -0.83438100 -0.16133100<br>N -3.62112600 -0.37155500 -0.46814200<br>N 2.07146100 1.09278700 -0.61317300<br>N 3.86117100 2.75800700 -0.23577800<br>C 0.05229300 -0.60064800 -1.30420500<br>C -1.27518000 -0.03112200 -0.86724700<br>C 1.90912300 0.03404400 0.15263500<br>C 0.57983300 -1.94591400 0.71466200<br>C -1.39296300 1.29000600 -0.42702500<br>C -2.42245800 -0.81377900 -0.86632400<br>C 2.74852500 -0.25697200 1.36330500<br>C -2.62515500 1.76455300 -0.01192200<br>C -3.69399300 0.87281700 -0.05916500<br>C 3.04030200 1.94967300 -0.37438800<br>H -0.10129200 -1.55877100 -1.80378600<br>H 0.55251800 0.07668000 -1.99233300<br>H -0.08018300 -1.61479800 1.52165100<br>H 1.46637100 -2.41030900 1.14055600<br>H 0.05699200 -2.69410900 0.12005800<br>H -0.52441400 1.94034000 -0.41778200	Zero-point correction= 0.203954 (Hartree/Particle)<br>Thermal correction to Energy= 0.218213<br>Thermal correction to Enthalpy= 0.219157<br>Thermal correction to Gibbs Free Energy= 0.160687<br>Sum of electronic and zero-point Energies= -1066.607481<br>Sum of electronic and thermal Energies= -1066.593222<br>Sum of electronic and thermal Enthalpies= -1066.592278<br>Sum of electronic and thermal Free Energies= -1066.650748

H -2.38474400 -1.84487700 -1.20621700<br>H 3.44074500 0.56040700 1.55206800<br>H 3.32174300 -1.17415200 1.21010900<br>H 2.11540000 -0.39641000 2.24130100<br>H -2.76246400 2.78133400 0.33094000	
**Name**	**AMP-W**
Cartesian Coordinates	Frequency and Energy
Cl -5.13181000 1.93356300 -0.12885900<br>N 0.96652900 -0.95214500 -0.07245500<br>N -3.55893000 0.26470200 -1.39232400<br>N 1.93402700 0.94354500 -0.89404700<br>N 3.58358100 2.78640200 -0.83020800<br>C 0.00120900 -0.93237500 -1.16518800<br>C -1.26446000 -0.17962600 -0.81607200<br>C 1.86098600 0.03043400 0.06021600<br>C 0.68093300 -1.87284300 1.03073400<br>C -1.35572400 0.71857200 0.24370400<br>C -2.39996000 -0.37028000 -1.59630500<br>C 2.72273100 0.03231500 1.28373600<br>C -2.55037900 1.38581600 0.47467000<br>C -3.60388300 1.11038000 -0.38421700<br>C 2.83122100 1.89833400 -0.80788700<br>H -0.24574900 -1.96511200 -1.41316200<br>H 0.46859200 -0.48206200 -2.03932500<br>H 0.13811500 -1.36768900 1.83381400<br>H 1.60097100 -2.29719000 1.42759100<br>H 0.06643300 -2.68201800 0.64248600<br>H -0.50866400 0.90250800 0.89629700<br>H -2.37847900 -1.06881300 -2.42674500<br>H 3.38814600 0.89250400 1.28635500<br>H 3.32008300 -0.88090000 1.32175000<br>H 2.09609100 0.06153800 2.17794200<br>H -2.66160900 2.09043800 1.28749900	Zero-point correction= 0.203603 (Hartree/Particle)<br>Thermal correction to Energy= 0.217914<br>Thermal correction to Enthalpy= 0.218858<br>Thermal correction to Gibbs Free Energy= 0.160139<br>Sum of electronic and zero-point Energies= -1066.602724<br>Sum of electronic and thermal Energies= -1066.588413<br>Sum of electronic and thermal Enthalpies= -1066.587469<br>Sum of electronic and thermal Free Energies= -1066.646188
**Name**	**RC-G**
Cartesian Coordinates	Frequency and Energy
Cl -5.65479400 1.06897200 -0.00155400<br>N 0.66396700 -0.99162200 -0.11465500<br>N -3.81097800 -0.40574600 -1.11688800<br>N 1.67860000 1.01862200 0.20857400<br>N 3.31061100 2.49994100 1.33779700<br>C -0.03978300 -0.45866300 -1.28659300<br>C -1.44252300 -0.01981000 -0.95028200<br>C 1.54156600 -0.23500000 0.56350000<br>C 0.33206300 -2.36345300 0.24385300<br>C -1.68038300 1.10842300 -0.15834400<br>C -2.54635000 -0.73160200 -1.39993900<br>C 2.30741700 -0.87116500 1.69251300<br>C -2.98236400 1.46164800 0.14256800<br>C -4.00127400 0.65541800 -0.36944900<br>C 2.56184700 1.77769100 0.83284900<br>H -0.06630100 -1.25111200 -2.03924700<br>H 0.54337000 0.37269200 -1.67784600<br>H -0.73357400 -2.51737600 0.06533700<br>H 0.52241600 -2.55585300 1.29599200	Zero-point correction= 0.214643 (Hartree/Particle)<br>Thermal correction to Energy= 0.232039<br>Thermal correction to Enthalpy= 0.232983<br>Thermal correction to Gibbs Free Energy= 0.163942<br>Sum of electronic and zero-point Energies= -1142.306685<br>Sum of electronic and thermal Energies= -1142.289290<br>Sum of electronic and thermal Enthalpies= -1142.288345<br>Sum of electronic and thermal Free Energies= -1142.357386

H 0.89910600 -3.07549200 -0.36336800<br>H -0.84555500 1.69797300 0.20549900<br>H -2.41607100 -1.60996200 -2.02750300<br>H 3.12301500 -0.22044000 2.00110300<br>H 2.71255500 -1.83841600 1.39515800<br>H 1.64854800 -1.02265600 2.55112200<br>H -3.21877400 2.32748900 0.74559000<br>O 5.40672800 3.73733300 2.96436200<br>H 4.69586900 3.38349700 2.38908400	
**Name**	**RC-P**
Cartesian Coordinates	Frequency and Energy
Cl -5.66190600 1.13777400 -0.09902300<br>N 0.65035300 -1.00231100 -0.07109700<br>N -3.79732500 -0.29200600 -1.24307000<br>N 1.73810300 0.96617000 0.26973300<br>N 3.38702200 2.43048900 1.37425400<br>C -0.02687500 -0.44710800 -1.24851300<br>C -1.43204300 0.00070500 -0.93142300<br>C 1.55311100 -0.29680700 0.60910700<br>C 0.31505600 -2.38632600 0.25231500<br>C -1.67940800 1.06305000 -0.05755700<br>C -2.52907500 -0.63818800 -1.49392800<br>C 2.29267300 -0.97369600 1.72645800<br>C -2.98457100 1.43460100 0.21291300<br>C -3.99241100 0.70793900 -0.41713200<br>C 2.62709800 1.70316900 0.88489700<br>H -0.04829900 -1.22950800 -2.01065000<br>H 0.56522000 0.38551300 -1.62294100<br>H -0.72600900 -2.55818000 -0.02234300<br>H 0.41592400 -2.57817000 1.31741900<br>H 0.94983500 -3.07801600 -0.30843500<br>H -0.85244300 1.59632800 0.39955300<br>H -2.38954700 -1.46700600 -2.18217600<br>H 3.12538600 -0.35728600 2.05857700<br>H 2.67191400 -1.94485100 1.40708100<br>H 1.61943200 -1.13114000 2.57308200<br>H -3.22124300 2.25322000 0.87912900<br>O 5.30400800 4.10542400 2.66057100<br>H 4.63497300 3.53754700 2.21243200	Zero-point correction= 0.214483 (Hartree/Particle)<br>Thermal correction to Energy= 0.230817<br>Thermal correction to Enthalpy= 0.231762<br>Thermal correction to Gibbs Free Energy= 0.166668<br>Sum of electronic and zero-point Energies= -1142.339770<br>Sum of electronic and thermal Energies= -1142.323436<br>Sum of electronic and thermal Enthalpies= -1142.322492<br>Sum of electronic and thermal Free Energies= -1142.387586
**Name**	**RC-W**
Cartesian Coordinates	Frequency and Energy
Cl -4.78231100 2.44822100 0.21663700<br>N 0.87806400 -1.22792900 -0.12452400<br>N -3.31799900 0.95198000 -1.35518200<br>N 1.90015700 0.77208100 -0.49843400<br>N 3.50821000 2.56472700 0.06504800<br>C -0.06135600 -0.92164700 -1.19770000<br>C -1.22480800 -0.06648100 -0.74153000<br>C 1.78371500 -0.32055600 0.24274800<br>C 0.54880300 -2.36845100 0.73388000<br>C -1.32776000 0.48719300 0.53093600<br>C -2.25173300 0.19865200 -1.64200800<br>C 2.60532500 -0.59088400 1.46372700<br>C -2.42699400 1.27304000 0.84947300	Zero-point correction= 0.213035 (Hartree/Particle)<br>Thermal correction to Energy= 0.230076<br>Thermal correction to Enthalpy= 0.231020<br>Thermal correction to Gibbs Free Energy= 0.165306<br>Sum of electronic and zero-point Energies= -1142.335640<br>Sum of electronic and thermal Energies= -1142.318599<br>Sum of electronic and thermal Enthalpies= -1142.317655<br>Sum of electronic and thermal Free Energies= -1142.383369

C -3.37761100 1.46095900 -0.14186600	
C 2.78067100 1.68428800 -0.16410000	
H -0.43494000 -1.86665000 -1.59185200	
H 0.47000500 -0.41401700 -2.00161000	
H 0.01123000 -3.09961700 0.13446300	
H -0.08249400 -2.05797400 1.57078400	
H 1.45345100 -2.83491900 1.11668000	
H -0.56476200 0.31766500 1.28346100	
H -2.21529900 -0.21405400 -2.64522000	
H 3.27194800 0.24264600 1.67340100	
H 3.19997300 -1.49519100 1.31788900	
H 1.95082800 -0.75196200 2.32314200	
H -2.54065400 1.72187900 1.82703500	
O 0.96897300 2.29978900 2.08880800	
H 1.78632300 2.74853900 1.79514900	
**Name**	**C2-RAF-G**
Cartesian Coordinates	Frequency and Energy
Cl -5.15574700 1.65785300 0.69673000	Zero-point correction= 0.214752 (Hartree/Particle)
N 0.85818400 -0.96926700 -0.25382700	Thermal correction to Energy= 0.231070
N -3.65832600 -0.29313000 -0.18352900	Thermal correction to Enthalpy= 0.232014
N 1.91341300 0.97666900 -0.77875500	Thermal correction to Gibbs Free Energy= 0.167605
N 3.48559700 2.86338400 -0.56596100	Sum of electronic and zero-point Energies= -1142.291761
C -0.09343600 -0.74080500 -1.34545000	Sum of electronic and thermal Energies= -1142.275443
C -1.35802300 -0.07714500 -0.85737900	Sum of electronic and thermal Enthalpies= -1142.274499
C 1.82568900 -0.06551600 0.00240200	Sum of electronic and thermal Free Energies= -1142.338908
C 0.51611400 -2.03178200 0.68233900	
C -1.39970600 1.28985400 -0.56441400	ν = -650.47
C -2.51844700 -0.81149300 -0.64925100	
C 2.71815500 -0.31353600 1.18823000	
C -2.57236100 1.84441900 -0.08436300	
C -3.66343600 0.99125100 0.08543400	
C 2.93088100 1.82107700 -0.65367600	
H -0.32742900 -1.71239400 -1.78608300	
H 0.40190400 -0.12809700 -2.09548400	
H -0.14057000 -1.67152200 1.48094100	
H 1.41105900 -2.46836100 1.11964000	
H -0.00748000 -2.81512400 0.13475300	
H -0.51723400 1.90104200 -0.71876100	
H -2.54287200 -1.87523000 -0.87303200	
H 3.37669700 0.53594900 1.34412300	
H 3.34092100 -1.19259400 1.01111700	
H 2.11742800 -0.48155400 2.08365600	
H -2.65672200 2.89648300 0.15062400	
O 4.50027200 0.72684300 -0.88313800	
H 5.21955500 1.35975000 -1.03012700	
**Name**	**C4-RAF-G**
Cartesian Coordinates	Frequency and Energy
Cl -5.19131800 1.46661500 0.52109000	Zero-point correction= 0.215593 (Hartree/Particle)
N 0.92487400 -0.89902300 -0.21521900	Thermal correction to Energy= 0.231311
N -3.61507400 -0.34418500 -0.50219200	Thermal correction to Enthalpy= 0.232255
N 2.00502500 1.08681300 -0.64964100	Thermal correction to Gibbs Free Energy= 0.170412
N 3.63176000 2.88369500 -0.10770000	Sum of electronic and zero-point Energies= -1142.280912
C 0.04711200 -0.65210800 -1.36036700	Sum of electronic and thermal Energies= -1142.265194
C -1.26049300 -0.06495400 -0.88565700	Sum of electronic and thermal Enthalpies= -1142.264250

C 2.02645000 -0.03882600 0.05013700	Sum of electronic and thermal Free Energies= -1142.326093
C 0.61744800 -2.01850600 0.65841200	
C -1.32465700 1.23814800 -0.38149700	ν =-463.47
C -2.43463500 -0.80499400 -0.92226300	
C 2.64946600 -0.13095900 1.41736200	
C -2.54059000 1.72969600 0.05547600	
C -3.64615500 0.88086700 -0.03077400	
C 2.89007800 2.02392000 -0.33706000	
H -0.11907500 -1.60711300 -1.86332400	
H 0.56014300 0.03648900 -2.02763600	
H 0.39706500 -1.66266100 1.66692700	
H 1.46737300 -2.70173700 0.68423800	
H -0.25588600 -2.53588900 0.26664200	
H -0.42587800 1.84544900 -0.34577200	
H -2.43709400 -1.81819400 -1.31676400	
H 3.61792600 0.36595100 1.37675300	
H 2.79488100 -1.15849000 1.73934000	
H 2.01817800 0.40314000 2.13249800	
H -2.64721500 2.73170200 0.44774600	
O 2.93980900 -1.33096100 -0.80368000	
H 2.97697600 -0.94178600 -1.69068400	
**Name**	**C5-FHT-G**
Cartesian Coordinates	Frequency and Energy
Cl -5.33704800 1.37482700 0.43136000	Zero-point correction= 0.210207 (Hartree/Particle)
N 0.86998300 -0.95779300 -0.20257700	Thermal correction to Energy= 0.226357
N -3.72660400 -0.16487700 -0.93175100	Thermal correction to Enthalpy= 0.227301
N 2.05194600 0.97755100 -0.50666500	Thermal correction to Gibbs Free Energy= 0.163200
N 3.71312800 2.59264300 0.38225000	Sum of electronic and zero-point Energies= -1142.295849
C 0.00316900 -0.60639700 -1.32916700	Sum of electronic and thermal Energies= -1142.279699
C -1.32507300 -0.06761200 -0.85401600	Sum of electronic and thermal Enthalpies= -1142.278754
C 1.84931700 -0.12130900 0.18251200	Sum of electronic and thermal Free Energies= -1142.342856
C 0.47319600 -2.11785000 0.59291800	
C -1.39566900 0.97831400 0.07123900	ν =-1482.54
C -2.52177500 -0.59901300 -1.31384600	
C 2.64372600 -0.43738500 1.40177200	
C -2.63338900 1.43923900 0.47864400	
C -3.76006200 0.82044700 -0.06660100	
C 2.95803200 1.82053100 -0.03866700	
H -0.15006300 -1.50288200 -1.93447600	
H 0.52654100 0.13185900 -1.93379600	
H -0.40406000 -2.56007900 0.12433800	
H 0.19952500 -1.83187200 1.61046000	
H 1.26406800 -2.86936200 0.62078800	
H -0.48807600 1.42583800 0.46438500	
H -2.52155700 -1.41813300 -2.02840600	
H 3.70033500 -0.21681500 1.26086000	
H 2.48267800 -1.41171200 1.84980500	
H 2.28110500 0.36225400 2.23113700	
H -2.73984000 2.24588000 1.19069400	
O 1.98507300 1.48986500 2.86454700	
H 2.58904100 2.11074100 2.41279200	
**Name**	**C7-FHT-G**
Cartesian Coordinates	Frequency and Energy
Cl -5.37235400 1.54941600 0.03532300	Zero-point correction= 0.210734 (Hartree/Particle)

N 0.85522300 -0.77023700 0.10375300<br>N -3.69725000 -0.33145800 -0.65599600<br>N 1.99046500 1.11273200 -0.48348500<br>N 3.83503600 2.75570800 -0.21465900<br>C 0.03566400 -0.67517800 -1.10937800<br>C -1.31312400 -0.06549800 -0.82627100<br>C 1.88234100 0.09045500 0.31515300<br>C 0.60275600 -1.88525900 0.96366000<br>C -1.43354900 1.27535600 -0.44726400<br>C -2.47787900 -0.81477900 -0.91422000<br>C 2.81109100 -0.20612200 1.45840300<br>C -2.68654100 1.79256100 -0.17833700<br>C -3.77696200 0.92862200 -0.30144500<br>C 2.99355500 1.96839100 -0.31116300<br>H -0.07882700 -1.68655700 -1.50781200<br>H 0.58516200 -0.07817200 -1.83449100<br>H 0.78178800 -1.65837400 2.01224200<br>H 1.31444100 -2.73808000 0.67395000<br>H -0.42019300 -2.23393000 0.82540900<br>H -0.54657600 1.89645200 -0.37513100<br>H -2.43905000 -1.85922500 -1.21364900<br>H 3.68352400 0.44157500 1.40173200<br>H 3.12493500 -1.25093100 1.42505800<br>H 2.30714400 -0.02012600 2.41034200<br>H -2.83363400 2.82360800 0.11238400<br>O 2.57426300 -3.43859500 0.16322000<br>H 2.45009800 -4.33558600 0.51453800	Thermal correction to Energy= 0.227493<br>Thermal correction to Enthalpy= 0.228437<br>Thermal correction to Gibbs Free Energy= 0.162217<br>Sum of electronic and zero-point Energies= -1142.296720<br>Sum of electronic and thermal Energies= -1142.279961<br>Sum of electronic and thermal Enthalpies= -1142.279017<br>Sum of electronic and thermal Free Energies= -1142.345237<br><br>ν =-744.10
**Name**	**C8-FHT-G**
Cartesian Coordinates	Frequency and Energy
Cl -4.83444200 2.20105600 0.58640100<br>N 0.96964600 -1.04760200 -0.19326700<br>N -3.30054100 0.10189100 0.33738200<br>N 1.51132600 1.15168700 -0.13050100<br>N 2.71164500 3.11971000 0.79550900<br>C 0.01057800 -0.74483000 -1.23279200<br>C -1.18417000 0.06949500 -0.80374000<br>C 1.67225200 -0.03351200 0.37358300<br>C 1.09624800 -2.42729500 0.26199900<br>C -1.32140800 1.42258100 -1.11354600<br>C -2.20738900 -0.53659200 -0.08389700<br>C 2.58347900 -0.35649800 1.52491600<br>C -2.45261600 2.09897100 -0.69092400<br>C -3.39885400 1.37696600 0.03465600<br>C 2.17267800 2.18055100 0.38949300<br>H -0.30787500 -1.68881500 -1.67563900<br>H 0.56450300 -0.15065500 -2.07128100<br>H 0.43141000 -2.63468200 1.10645000<br>H 2.12082600 -2.64826600 0.55367700<br>H 0.83318000 -3.09141800 -0.55984200<br>H -0.53921700 1.92403100 -1.67172200<br>H -2.15308700 -1.59313000 0.16890600<br>H 2.93057800 0.56517100 1.98767900<br>H 3.45479900 -0.91238400 1.16996700<br>H 2.07002200 -0.96372400 2.27140800	Zero-point correction= 0.209566 (Hartree/Particle)<br>Thermal correction to Energy= 0.226493<br>Thermal correction to Enthalpy= 0.227437<br>Thermal correction to Gibbs Free Energy= 0.161898<br>Sum of electronic and zero-point Energies= -1142.291387<br>Sum of electronic and thermal Energies= -1142.274460<br>Sum of electronic and thermal Enthalpies= -1142.273516<br>Sum of electronic and thermal Free Energies= -1142.339055<br><br>ν =-741.06

H -2.60570200 3.14840600 -0.90112300<br>O 1.00606500 0.43893500 -3.30273500<br>H 0.38166800 0.02957700 -3.92406200	
**Name**	**C9-RAF-G**
Cartesian Coordinates	Frequency and Energy
Cl -4.74493200 1.71419300 1.07381600<br>N 0.93727200 -0.92844500 -0.27912300<br>N -3.30803200 -0.24126300 0.11830900<br>N 1.80537000 1.17250800 -0.40203000<br>N 3.31215700 2.98951800 0.37263600<br>C 0.10249300 -0.63246200 -1.44493500<br>C -1.20154300 0.02991600 -1.05356700<br>C 1.74718700 0.02951600 0.22324100<br>C 0.63263000 -2.14640400 0.46024000<br>C -1.27989400 1.43036700 -0.84588000<br>C -2.25051800 -0.74621100 -0.50146200<br>C 2.53227600 -0.29041600 1.46669200<br>C -2.38014300 1.96146700 -0.21019700<br>C -3.35769600 1.07162200 0.25110200<br>C 2.62809100 2.11792100 0.04016000<br>H -0.11254000 -1.56988700 -1.95732800<br>H 0.65221700 0.01677900 -2.12163500<br>H -0.14816600 -1.98274900 1.21120100<br>H 1.52062800 -2.53695700 0.95236200<br>H 0.28258900 -2.89923900 -0.24523400<br>H -0.47851800 2.06246800 -1.20716900<br>H -2.23313700 -1.82594000 -0.62138700<br>H 3.11091900 0.57763100 1.77471200<br>H 3.21682500 -1.12048200 1.28040800<br>H 1.86207900 -0.57769600 2.27894700<br>H -2.49316000 3.02516300 -0.05160200<br>O -1.85117300 -0.13774100 -2.95195300<br>H -2.64984600 0.41157800 -2.93571400	Zero-point correction= 0.214638 (Hartree/Particle)<br>Thermal correction to Energy= 0.231008<br>Thermal correction to Enthalpy= 0.231953<br>Thermal correction to Gibbs Free Energy= 0.168730<br>Sum of electronic and zero-point Energies= -1142.296320<br>Sum of electronic and thermal Energies= -1142.279949<br>Sum of electronic and thermal Enthalpies= -1142.279005<br>Sum of electronic and thermal Free Energies= -1142.342228<br><br>ν =-442.98
**Name**	**C10-RAF-G**
Cartesian Coordinates	Frequency and Energy
Cl -5.00813600 2.16348300 0.26380100<br>N 0.92758800 -0.82166100 -0.38760200<br>N -2.98986200 0.52536900 0.46476700<br>N 1.67366400 1.20499000 0.29828900<br>N 3.03396100 2.67823600 1.76528300<br>C 0.21496000 -0.12776400 -1.45195600<br>C -1.03742500 0.56071600 -0.98482700<br>C 1.68632200 -0.09007000 0.45428400<br>C 0.79973700 -2.27395000 -0.30837000<br>C -1.55304100 1.66746400 -1.64893400<br>C -1.80486500 0.00089300 0.06204600<br>C 2.47939800 -0.81272600 1.50826200<br>C -2.78073900 2.17534000 -1.26974900<br>C -3.45182900 1.53753100 -0.21009600<br>C 2.41328400 1.96238400 1.10159000<br>H -0.07833700 -0.87610800 -2.19369600<br>H 0.88072100 0.59822700 -1.92108800<br>H 0.89220400 -2.61240100 0.72185600<br>H 1.55903400 -2.77477000 -0.91711000	Zero-point correction= 0.214618 (Hartree/Particle)<br>Thermal correction to Energy= 0.231034<br>Thermal correction to Enthalpy= 0.231979<br>Thermal correction to Gibbs Free Energy= 0.168418<br>Sum of electronic and zero-point Energies= -1142.295626<br>Sum of electronic and thermal Energies= -1142.279210<br>Sum of electronic and thermal Enthalpies= -1142.278265<br>Sum of electronic and thermal Free Energies= -1142.341826<br><br>ν =-486.73

H -0.19310800 -2.54817100 -0.66530100<br>H -0.99382000 2.12969400 -2.45467500<br>H -1.35044400 -0.69572500 0.75468100<br>H 3.18375900 -0.12719300 1.97514000<br>H 3.02294700 -1.65463600 1.07840900<br>H 1.81002800 -1.19760000 2.28183200<br>H -3.22231100 3.03693900 -1.75102300<br>O -2.32956400 -1.56954600 -1.09913300<br>H -3.24373100 -1.60716200 -0.77833200	
**Name**	**C12-RAF-G**
Cartesian Coordinates	Frequency and Energy
Cl -4.98128500 1.49321000 0.84307200<br>N 0.96946900 -0.92088300 -0.19764200<br>N -3.50357200 -0.36093300 -0.26652100<br>N 1.95416600 1.10979900 -0.51136400<br>N 3.62551100 2.86199100 0.04551400<br>C 0.09701900 -0.65802900 -1.34673700<br>C -1.20581600 -0.02051100 -0.93019700<br>C 1.85509800 0.02101000 0.19909700<br>C 0.58257000 -2.03729500 0.65420900<br>C -1.31144900 1.34727200 -0.68482300<br>C -2.34538500 -0.81502000 -0.70138200<br>C 2.67372400 -0.25639800 1.43117300<br>C -2.52167700 1.86048600 -0.24958200<br>C -3.62108400 0.96962700 -0.11054700<br>C 2.86389700 2.02271700 -0.18502800<br>H -0.09392100 -1.61591600 -1.83563000<br>H 0.63738900 -0.01361600 -2.03690500<br>H -0.21368100 -1.75606800 1.35174400<br>H 1.43356800 -2.41254600 1.21675200<br>H 0.21746500 -2.84554400 0.02001300<br>H -0.45189200 1.99275100 -0.82179000<br>H -2.28898700 -1.89023700 -0.86162500<br>H 3.34453300 0.57730000 1.62650500<br>H 3.26742400 -1.16288900 1.29922600<br>H 2.02287000 -0.39714900 2.29628800<br>H -2.66877600 2.91214000 -0.04811000<br>O -4.28011400 1.61517800 -1.82841800<br>H -3.74895700 1.10158600 -2.45746200	Zero-point correction= 0.214425 (Hartree/Particle)<br>Thermal correction to Energy= 0.230844<br>Thermal correction to Enthalpy= 0.231788<br>Thermal correction to Gibbs Free Energy= 0.167783<br>Sum of electronic and zero-point Energies= -1142.283064<br>Sum of electronic and thermal Energies= -1142.266645<br>Sum of electronic and thermal Enthalpies= -1142.265700<br>Sum of electronic and thermal Free Energies= -1142.329706<br><br>ν =-612.25
**Name**	**C13-RAF-G**
Cartesian Coordinates	Frequency and Energy
Cl -4.99271200 1.61493500 0.78672600<br>N 0.99510000 -0.95523700 -0.23487800<br>N -3.43432900 -0.30958400 -0.02102600<br>N 1.88453000 1.13067400 -0.43855200<br>N 3.48495900 2.92031400 0.20191000<br>C 0.09064700 -0.65006300 -1.34796200<br>C -1.19309400 -0.00538700 -0.87909800<br>C 1.84586900 -0.00142700 0.20688600<br>C 0.66860700 -2.13342900 0.55616500<br>C -1.31809700 1.37229600 -0.74382400<br>C -2.28933000 -0.79210300 -0.52036200<br>C 2.69686400 -0.31508900 1.40787200<br>C -2.54268600 1.91615400 -0.31358000	Zero-point correction= 0.214637 (Hartree/Particle)<br>Thermal correction to Energy= 0.231173<br>Thermal correction to Enthalpy= 0.232117<br>Thermal correction to Gibbs Free Energy= 0.167787<br>Sum of electronic and zero-point Energies= -1142.291655<br>Sum of electronic and thermal Energies= -1142.275119<br>Sum of electronic and thermal Enthalpies= -1142.274174<br>Sum of electronic and thermal Free Energies= -1142.338504<br><br>ν =-533.69

C -3.53021800 0.98921400 0.10864600<br>C 2.75755200 2.06302200 -0.06918300<br>H -0.12695200 -1.59278900 -1.85427500<br>H 0.61663800 0.00427100 -2.03978600<br>H -0.12341600 -1.92543100 1.28384300<br>H 1.54462500 -2.50592900 1.08105700<br>H 0.32328500 -2.91800200 -0.11747000<br>H -0.49558400 2.02763600 -1.00303500<br>H -2.24727300 -1.87288600 -0.63147400<br>H 3.33593200 0.53350000 1.64176400<br>H 3.32522200 -1.18636900 1.21372100<br>H 2.06784900 -0.53336300 2.27308900<br>H -2.62524100 2.95365100 -0.02614200<br>O -3.33344800 2.43667200 -2.06917500<br>H -3.26683400 1.60075500 -2.55687800	
**Name**	**C14-RAF-G**
Cartesian Coordinates	Frequency and Energy
Cl -5.02512800 1.73219000 0.77613900<br>N 0.97674700 -0.93303200 -0.25500400<br>N -3.43712900 -0.21535400 0.05791200<br>N 1.80522100 1.18539100 -0.33719900<br>N 3.36833300 2.97408800 0.39200300<br>C 0.05314400 -0.58865000 -1.34213900<br>C -1.21664600 0.04367300 -0.83345500<br>C 1.81061700 0.01293500 0.23247100<br>C 0.70090600 -2.16915800 0.46396900<br>C -1.36468800 1.45834800 -0.82255400<br>C -2.29291600 -0.72186600 -0.41712800<br>C 2.69477400 -0.35465600 1.39382000<br>C -2.54649200 1.99022000 -0.26173000<br>C -3.53388900 1.09977900 0.12934300<br>C 2.65987400 2.11594000 0.07681100<br>H -0.17578600 -1.51285500 -1.87583400<br>H 0.55433800 0.09438200 -2.02528800<br>H -0.07531800 -2.03064900 1.22448200<br>H 1.59991100 -2.55206700 0.94054400<br>H 0.35707800 -2.91633800 -0.25128800<br>H -0.48758200 2.08177900 -0.93330800<br>H -2.24702800 -1.80676500 -0.46803200<br>H 3.31371800 0.49538400 1.67230000<br>H 3.34387500 -1.19160600 1.12949100<br>H 2.09068000 -0.64930100 2.25410800<br>H -2.69167700 3.05803200 -0.17370900<br>O -1.66929900 1.64877900 -2.73544300<br>H -2.59771200 1.37639800 -2.81410300	Zero-point correction= 0.214511 (Hartree/Particle)<br>Thermal correction to Energy= 0.230969<br>Thermal correction to Enthalpy= 0.231914<br>Thermal correction to Gibbs Free Energy= 0.167829<br>Sum of electronic and zero-point Energies= -1142.289200<br>Sum of electronic and thermal Energies= -1142.272741<br>Sum of electronic and thermal Enthalpies= -1142.271797<br>Sum of electronic and thermal Free Energies= -1142.335882<br><br>ν =-579.76
**Name**	**C2-RAF-P**
Cartesian Coordinates	Frequency and Energy
Cl -5.29359000 1.53524900 0.58638000<br>N 0.84365500 -0.88452400 -0.20692200<br>N -3.73237300 -0.32876300 -0.37186000<br>N 1.99936800 0.97808500 -0.82345300<br>N 3.63901900 2.80593800 -0.72584400<br>C -0.09548700 -0.69161300 -1.31618400<br>C -1.38931700 -0.07510400 -0.84400400	Zero-point correction= 0.214579 (Hartree/Particle)<br>Thermal correction to Energy= 0.230850<br>Thermal correction to Enthalpy= 0.231794<br>Thermal correction to Gibbs Free Energy= 0.167896<br>Sum of electronic and zero-point Energies= -1142.317855<br>Sum of electronic and thermal Energies= -1142.301585<br>Sum of electronic and thermal Enthalpies= -1142.300641

C 1.85834800 -0.03878700 -0.00018100<br>C 0.51031600 -1.93998000 0.74699900<br>C -1.44730600 1.25300300 -0.41251500<br>C -2.56223600 -0.81700300 -0.80057300<br>C 2.74611800 -0.29206300 1.18443800<br>C -2.64902200 1.77471400 0.03367300<br>C -3.74974600 0.92093100 0.02593000<br>C 3.04417300 1.77626100 -0.75287900<br>H -0.28859100 -1.66925900 -1.76153900<br>H 0.38272600 -0.05810500 -2.05971400<br>H -0.00114100 -1.53528100 1.62475000<br>H 1.40430900 -2.47548500 1.06193600<br>H -0.15452200 -2.64688300 0.25313900<br>H -0.55609100 1.87176900 -0.43456400<br>H -2.57094800 -1.85231400 -1.12914800<br>H 3.45431900 0.52179800 1.31075400<br>H 3.30593300 -1.21911700 1.04295000<br>H 2.14267400 -0.39171500 2.08870600<br>H -2.74002800 2.79845800 0.37118800<br>O 4.56744500 0.65204000 -1.00526900<br>H 5.33607600 1.24477400 -1.02943600	Sum of electronic and thermal Free Energies= -1142.364538<br><br>ν =-671.51
**Name**	**C4-RAF-P**
Cartesian Coordinates	Frequency and Energy
Cl -5.21401200 1.44054300 0.51504300<br>N 0.94074300 -0.88779700 -0.21116500<br>N -3.59668600 -0.36606200 -0.45546800<br>N 2.01169300 1.11273400 -0.59436200<br>N 3.64359500 2.90610100 -0.10876400<br>C 0.05728100 -0.62818000 -1.35263400<br>C -1.25443800 -0.04838500 -0.87653500<br>C 2.01201700 -0.03058800 0.08373600<br>C 0.61572600 -2.01278700 0.65488900<br>C -1.34690300 1.26910100 -0.42072100<br>C -2.41022800 -0.81920500 -0.87260400<br>C 2.66941600 -0.17255700 1.42594600<br>C -2.56878100 1.75290300 0.01358200<br>C -3.64901500 0.87491000 -0.03040700<br>C 2.89889800 2.03559900 -0.30205300<br>H -0.11029400 -1.58132500 -1.85666400<br>H 0.56049000 0.06092400 -2.02612100<br>H 0.31392700 -1.65216700 1.64109800<br>H 1.48060600 -2.66879700 0.74987900<br>H -0.20608300 -2.56783200 0.20725300<br>H -0.46846600 1.90535800 -0.41577600<br>H -2.38979100 -1.84550800 -1.22793100<br>H 3.65310700 0.29324200 1.38014700<br>H 2.78816500 -1.20962800 1.72568300<br>H 2.06626000 0.35715300 2.16875900<br>H -2.68885000 2.76745100 0.36917200<br>O 2.96357800 -1.33210700 -0.85436500<br>H 2.89454100 -0.97099400 -1.75322500	Zero-point correction= 0.215502 (Hartree/Particle)<br>Thermal correction to Energy= 0.231150<br>Thermal correction to Enthalpy= 0.232094<br>Thermal correction to Gibbs Free Energy= 0.170682<br>Sum of electronic and zero-point Energies= -1142.314090<br>Sum of electronic and thermal Energies= -1142.298442<br>Sum of electronic and thermal Enthalpies= -1142.297498<br>Sum of electronic and thermal Free Energies= -1142.358910<br><br>ν =-478.34
**Name**	**C5-FHT-P**
Cartesian Coordinates	Frequency and Energy
Cl -5.35142600 1.50049700 0.32913900	Zero-point correction= 0.209396 (Hartree/Particle)

N 0.88211900 -0.82657100 -0.10584700<br>N -3.72484600 -0.22764900 -0.76658600<br>N 2.05077500 1.07029100 -0.60569600<br>N 3.88294700 2.69415200 -0.25214400<br>C -0.00252300 -0.61463500 -1.25374800<br>C -1.33062200 -0.03439700 -0.83373900<br>C 1.90505100 -0.00540700 0.14169000<br>C 0.58595000 -1.99063900 0.72648800<br>C -1.40941300 1.17724300 -0.14241900<br>C -2.51930200 -0.69376400 -1.11456500<br>C 2.80753200 -0.33808900 1.28109400<br>C -2.64721500 1.67351900 0.22593100<br>C -3.76179000 0.91380200 -0.12204500<br>C 3.04283100 1.90501000 -0.38553900<br>H -0.15424000 -1.58126200 -1.73928900<br>H 0.50015900 0.04936500 -1.95443800<br>H -0.47306200 -2.22332500 0.61710600<br>H 0.77109400 -1.77956000 1.77790200<br>H 1.17408600 -2.85663600 0.41108500<br>H -0.50620800 1.72761800 0.10053000<br>H -2.51089500 -1.64174200 -1.64474300<br>H 3.69124400 0.29373900 1.31603800<br>H 3.06301900 -1.39476200 1.34853600<br>H 2.19723200 -0.12744200 2.27156300<br>H -2.75498300 2.60842900 0.75892400<br>O 1.42224800 0.34735200 3.30879300<br>H 1.75788500 1.26176100 3.33651100	Thermal correction to Energy= 0.226121<br>Thermal correction to Enthalpy= 0.227065<br>Thermal correction to Gibbs Free Energy= 0.160885<br>Sum of electronic and zero-point Energies= -1142.325342<br>Sum of electronic and thermal Energies= -1142.308617<br>Sum of electronic and thermal Enthalpies= -1142.307673<br>Sum of electronic and thermal Free Energies= -1142.373853<br><br>ν =-1130.33
**Name**	**C7-FHT-P**
Cartesian Coordinates	Frequency and Energy
Cl -5.36616900 1.50926600 0.06009300<br>N 0.89585900 -0.75679000 0.09236700<br>N -3.66170800 -0.35647500 -0.60650200<br>N 1.99690600 1.15136300 -0.47214300<br>N 3.76614600 2.86330100 -0.22144500<br>C 0.06418900 -0.65971800 -1.11514900<br>C -1.28636500 -0.05820500 -0.81874600<br>C 1.89389500 0.11532200 0.32496000<br>C 0.65914100 -1.89433400 0.93519500<br>C -1.42490400 1.28890000 -0.47297000<br>C -2.43867100 -0.83135700 -0.87100300<br>C 2.80730600 -0.15660600 1.48299000<br>C -2.68219900 1.79671700 -0.19714500<br>C -3.75424500 0.91129800 -0.28316700<br>C 2.95859200 2.03523500 -0.30100300<br>H -0.05219500 -1.67157000 -1.50911500<br>H 0.59912300 -0.06131700 -1.84939200<br>H 0.87905000 -1.69840400 1.98146400<br>H 1.33744800 -2.75267700 0.58960500<br>H -0.37069800 -2.23118000 0.82271300<br>H -0.55174100 1.93147100 -0.42929900<br>H -2.38451500 -1.88173500 -1.14302900<br>H 3.66597500 0.51047600 1.45167300<br>H 3.15496100 -1.19065200 1.45892700<br>H 2.27509300 0.00581000 2.42389900	Zero-point correction= 0.210598 (Hartree/Particle)<br>Thermal correction to Energy= 0.227234<br>Thermal correction to Enthalpy= 0.228178<br>Thermal correction to Gibbs Free Energy= 0.162810<br>Sum of electronic and zero-point Energies= -1142.328449<br>Sum of electronic and thermal Energies= -1142.311813<br>Sum of electronic and thermal Enthalpies= -1142.310869<br>Sum of electronic and thermal Free Energies= -1142.376237<br><br>ν =-770.80

H -2.83608800 2.83400800 0.06841800	
O 2.39626500 -3.69808400 0.03613900	
H 2.21109400 -4.46729400 0.60353300	
**Name**	**C8-FHT-P**
Cartesian Coordinates	Frequency and Energy
Cl -4.83730400 2.22695400 0.62522900	Zero-point correction= 0.209444 (Hartree/Particle)
N 0.94344500 -1.03554700 -0.18695200	Thermal correction to Energy= 0.226395
N -3.22400800 0.17639200 0.47024300	Thermal correction to Enthalpy= 0.227339
N 1.46334100 1.16381600 -0.04343300	Thermal correction to Gibbs Free Energy= 0.161760
N 2.61964800 3.13712200 0.90120800	Sum of electronic and zero-point Energies= -1142.325568
C 0.00780900 -0.70264300 -1.23694900	Sum of electronic and thermal Energies= -1142.308616
C -1.18633500 0.10949300 -0.79889300	Sum of electronic and thermal Enthalpies= -1142.307672
C 1.64138700 -0.05037400 0.41121600	Sum of electronic and thermal Free Energies= -1142.373251
C 1.12006800 -2.43657000 0.19949000	
C -1.39765000 1.42082800 -1.21807100	ν =-785.78
C -2.13205900 -0.46440200 0.04339600	
C 2.56717200 -0.41912600 1.53001400	
C -2.52878300 2.09767700 -0.79074700	
C -3.39412700 1.41118700 0.05429700	
C 2.10301400 2.18348500 0.49145700	
H -0.31029700 -1.62887800 -1.71343700	
H 0.57396700 -0.08848800 -2.04644100	
H 0.63653900 -2.64136100 1.15800800	
H 2.17759500 -2.69013200 0.26719200	
H 0.66470600 -3.06461000 -0.56269200	
H -0.67474200 1.90370900 -1.86602600	
H -2.01065200 -1.48542900 0.39551800	
H 2.93707100 0.47544400 2.02641500	
H 3.42071100 -0.97719200 1.13669400	
H 2.05830500 -1.05034500 2.26010100	
H -2.73190700 3.11753000 -1.08817100	
O 1.07863700 0.45842600 -3.29804400	
H 0.63005000 -0.14734000 -3.91366700	
**Name**	**C9-RAF-P**
Cartesian Coordinates	Frequency and Energy
Cl -4.73693000 1.76647500 1.06202500	Zero-point correction= 0.214573 (Hartree/Particle)
N 0.92692300 -0.91961300 -0.26230600	Thermal correction to Energy= 0.230908
N -3.28082100 -0.20456000 0.16749400	Thermal correction to Enthalpy= 0.231852
N 1.79725000 1.17809800 -0.36196000	Thermal correction to Gibbs Free Energy= 0.168499
N 3.27097000 3.01695100 0.39117200	Sum of electronic and zero-point Energies= -1142.327495
C 0.10353300 -0.62024100 -1.43762200	Sum of electronic and thermal Energies= -1142.311160
C -1.20115900 0.04356600 -1.05053200	Sum of electronic and thermal Enthalpies= -1142.310216
C 1.73578000 0.01591900 0.25136900	Sum of electronic and thermal Free Energies= -1142.373569
C 0.67962900 -2.18558400 0.42529100	
C -1.29698600 1.45011400 -0.90325200	ν =-430.87
C -2.22670600 -0.72633500 -0.44643100	
C 2.51337200 -0.32048700 1.49060300	
C -2.39332600 1.99682800 -0.27203800	
C -3.34508900 1.11254200 0.24164100	
C 2.60019900 2.12470200 0.07505300	
H -0.10570100 -1.56002800 -1.94594300	
H 0.65898100 0.02961300 -2.10969200	
H -0.05659000 -2.06645600 1.22607800	
H 1.60080500 -2.58531800 0.84397200	

H 0.29946900 -2.90256800 -0.30013200<br>H -0.51763400 2.08121600 -1.31197900<br>H -2.18863700 -1.80999100 -0.50815700<br>H 3.06508300 0.54870900 1.84154500<br>H 3.22223400 -1.12532300 1.28292100<br>H 1.84094300 -0.65751300 2.28169600<br>H -2.51692400 3.06577600 -0.16247400<br>O -1.90577300 -0.17194300 -2.93410500<br>H -2.68945900 0.40020600 -2.89962900	
**Name**	**C10-RAF-P**
Cartesian Coordinates	Frequency and Energy
Cl -5.20890300 1.52748100 0.60559800<br>N 0.92958900 -0.83032700 -0.17624100<br>N -3.62596800 -0.29977300 -0.36680500<br>N 2.01977900 1.13434900 -0.55128500<br>N 3.78474600 2.81341400 -0.12017900<br>C 0.03942200 -0.56654900 -1.31084500<br>C -1.26688000 0.02931500 -0.85380100<br>C 1.88432800 0.04193700 0.16986900<br>C 0.58597100 -1.98056900 0.65637000<br>C -1.36780400 1.35946300 -0.43431300<br>C -2.44434000 -0.75360700 -0.86678800<br>C 2.73811100 -0.28507000 1.36081100<br>C -2.58691900 1.84260400 -0.00407100<br>C -3.67064800 0.94207600 0.00878900<br>C 2.97717000 1.99714000 -0.28579500<br>H -0.14519600 -1.51288100 -1.82003900<br>H 0.54355900 0.10918400 -1.99835900<br>H -0.09372700 -1.69459800 1.46440000<br>H 1.47851000 -2.43438100 1.08063100<br>H 0.09384400 -2.72186200 0.02829000<br>H -0.49295900 1.99995500 -0.44977600<br>H -2.38005300 -1.82543600 -1.00832400<br>H 3.42134800 0.53344800 1.57550100<br>H 3.32141600 -1.18791800 1.16600700<br>H 2.11508500 -0.46746400 2.23813200<br>H -2.71630500 2.86414200 0.32753200<br>O -2.56485400 -0.55794900 -2.84651000<br>H -2.82792700 0.37565900 -2.90044900	Zero-point correction= 0.214889 (Hartree/Particle)<br>Thermal correction to Energy= 0.231263<br>Thermal correction to Enthalpy= 0.232207<br>Thermal correction to Gibbs Free Energy= 0.168504<br>Sum of electronic and zero-point Energies= -1142.324050<br>Sum of electronic and thermal Energies= -1142.307676<br>Sum of electronic and thermal Enthalpies= -1142.306732<br>Sum of electronic and thermal Free Energies= -1142.370435<br><br>ν =-514.91
**Name**	**C12-RAF-P**
Cartesian Coordinates	Frequency and Energy
Cl -5.28785900 1.19862000 0.51179600<br>N 0.93134300 -0.83575300 -0.09117700<br>N -3.66872300 -0.43432700 -0.73936200<br>N 2.07247400 1.03940200 -0.70290500<br>N 3.86804200 2.72421900 -0.46285600<br>C 0.05576100 -0.70538100 -1.25678200<br>C -1.28273400 -0.11876100 -0.88433800<br>C 1.90738200 0.04896800 0.14746100<br>C 0.57218600 -1.87448900 0.87213900<br>C -1.39075400 1.14242200 -0.30302100<br>C -2.46420700 -0.86173500 -1.06749500<br>C 2.74317200 -0.14894600 1.37870000<br>C -2.64254100 1.62541300 0.03661500	Zero-point correction= 0.214419 (Hartree/Particle)<br>Thermal correction to Energy= 0.230795<br>Thermal correction to Enthalpy= 0.231740<br>Thermal correction to Gibbs Free Energy= 0.168061<br>Sum of electronic and zero-point Energies= -1142.317623<br>Sum of electronic and thermal Energies= -1142.301246<br>Sum of electronic and thermal Enthalpies= -1142.300302<br>Sum of electronic and thermal Free Energies= -1142.363980<br><br>ν =-604.61

C -3.76943900 0.82028500 -0.27348900<br>C 3.04451700 1.91043900 -0.53610300<br>H -0.07999800 -1.69930700 -1.68705500<br>H 0.55441100 -0.07318900 -1.98959100<br>H -0.02522900 -1.46543200 1.69168300<br>H 1.46077900 -2.35545200 1.27663900<br>H -0.01748200 -2.62965600 0.35438100<br>H -0.50567100 1.73965400 -0.11240100<br>H -2.40558700 -1.86750600 -1.47570000<br>H 3.44328300 0.67421700 1.50151000<br>H 3.30690200 -1.08152900 1.30249500<br>H 2.10736000 -0.21009900 2.26376000<br>H -2.78612100 2.59699500 0.48936800<br>O -4.09251800 1.90495200 -1.88523500<br>H -3.34064300 1.65800800 -2.44920700	
**Name**	**C13-RAF-P**
Cartesian Coordinates	Frequency and Energy
Cl -4.91172200 1.79585000 0.84537000<br>N 0.98138500 -0.96111000 -0.22800000<br>N -3.35742800 -0.18365400 0.16809700<br>N 1.82095200 1.14756000 -0.33474600<br>N 3.32558400 2.98359300 0.36268100<br>C 0.08156400 -0.62999400 -1.33929000<br>C -1.18157400 0.04757600 -0.85981400<br>C 1.80766500 -0.02687500 0.25838700<br>C 0.76892900 -2.23522800 0.45454200<br>C -1.32943800 1.42683100 -0.88072300<br>C -2.23500600 -0.71058700 -0.34185200<br>C 2.65613300 -0.37786800 1.44567400<br>C -2.53534600 2.00541000 -0.43745300<br>C -3.47069300 1.11945900 0.14878500<br>C 2.64213300 2.09273900 0.07105100<br>H -0.16157800 -1.56336000 -1.84797400<br>H 0.61322600 0.01724900 -2.03407000<br>H 0.06588700 -2.12893000 1.28620200<br>H 1.70926200 -2.63327600 0.82981900<br>H 0.36297400 -2.94680100 -0.26228800<br>H -0.54102400 2.06137700 -1.26708400<br>H -2.17428200 -1.79514100 -0.32629100<br>H 3.23065600 0.48620900 1.77178500<br>H 3.34789800 -1.18367400 1.19068800<br>H 2.02861800 -0.71896100 2.27141000<br>H -2.61357500 3.06854700 -0.26308200<br>O -3.47718900 2.29021200 -2.19098900<br>H -3.33554700 1.41885200 -2.59585400	Zero-point correction= 0.214785 (Hartree/Particle)<br>Thermal correction to Energy= 0.231154<br>Thermal correction to Enthalpy= 0.232098<br>Thermal correction to Gibbs Free Energy= 0.168768<br>Sum of electronic and zero-point Energies= -1142.324947<br>Sum of electronic and thermal Energies= -1142.308578<br>Sum of electronic and thermal Enthalpies= -1142.307634<br>Sum of electronic and thermal Free Energies= -1142.370964<br><br>ν =-501.87
**Name**	**C14-RAF-P**
Cartesian Coordinates	Frequency and Energy
Cl -5.26648900 1.43849600 0.51507600<br>N 0.92628300 -0.84350400 -0.15981100<br>N -3.62686600 -0.37306500 -0.41506600<br>N 2.01590600 1.10969000 -0.59538700<br>N 3.78268900 2.79850500 -0.21215900<br>C 0.03539600 -0.62168800 -1.30385600<br>C -1.28819600 -0.05632400 -0.86709400	Zero-point correction= 0.214387 (Hartree/Particle)<br>Thermal correction to Energy= 0.230859<br>Thermal correction to Enthalpy= 0.231804<br>Thermal correction to Gibbs Free Energy= 0.167519<br>Sum of electronic and zero-point Energies= -1142.321381<br>Sum of electronic and thermal Energies= -1142.304909<br>Sum of electronic and thermal Enthalpies= -1142.303965

C 1.88166000 0.03953100 0.15753000<br>C 0.59578000 -1.97487400 0.70328600<br>C -1.39967900 1.31956500 -0.52362300<br>C -2.43208500 -0.83192800 -0.81889600<br>C 2.73774500 -0.25303400 1.35602400<br>C -2.63881500 1.78043500 -0.02968700<br>C -3.69766500 0.88672400 -0.03510600<br>C 2.97299300 1.98013900 -0.35550500<br>H -0.11197400 -1.58247000 -1.79943700<br>H 0.51961100 0.06601800 -1.99488100<br>H -0.06723100 -1.67127000 1.51845500<br>H 1.49565600 -2.42303800 1.11842400<br>H 0.08857600 -2.72788400 0.10145700<br>H -0.50243300 1.89978000 -0.34619900<br>H -2.40011200 -1.87616800 -1.11478500<br>H 3.41471800 0.57552600 1.55119100<br>H 3.32806600 -1.15556400 1.18178000<br>H 2.11677300 -0.42016100 2.23778400<br>H -2.76336200 2.80212600 0.30304200<br>O -1.43088100 2.05288600 -2.33979100<br>H -2.31813700 1.78657300 -2.63558500	Sum of electronic and thermal Free Energies= -1142.368249<br><br>ν =-580.44
**Name**	**C2-RAF-W**
Cartesian Coordinates	Frequency and Energy
Cl -5.23685600 1.86251200 0.12019900<br>N 0.88052200 -0.94743400 -0.14878900<br>N -3.72652200 0.17355900 -1.19059500<br>N 1.91230200 0.87355200 -1.05835100<br>N 3.48439100 2.76352600 -1.16505500<br>C -0.12577600 -0.91849600 -1.20471300<br>C -1.38098600 -0.18447800 -0.78866800<br>C 1.83553500 -0.02052700 -0.08463200<br>C 0.60740700 -1.83936400 0.98134100<br>C -1.40933500 0.76360200 0.23102500<br>C -2.57076400 -0.44306000 -1.45999800<br>C 2.75045200 -0.03804800 1.10064600<br>C -2.59924100 1.41172800 0.52922400<br>C -3.71203500 1.06731300 -0.22430800<br>C 2.91112400 1.72259700 -1.08165200<br>H -0.37411700 -1.94898700 -1.46103900<br>H 0.30539400 -0.44820900 -2.08653200<br>H 0.14086300 -1.29549400 1.80644400<br>H 1.52409100 -2.31114700 1.32988200<br>H -0.07337900 -2.61544000 0.63852000<br>H -0.51613600 0.99965900 0.80046100<br>H -2.59782100 -1.18307700 -2.25349600<br>H 3.41375900 0.82350900 1.08947000<br>H 3.35021100 -0.95102300 1.09604200<br>H 2.16320300 -0.02443900 2.02088900<br>H -2.66373500 2.15221600 1.31488500<br>O 4.52828800 0.68167900 -1.39160900<br>H 5.18324300 1.09079600 -0.80283700	Zero-point correction= 0.213914 (Hartree/Particle)<br>Thermal correction to Energy= 0.230436<br>Thermal correction to Enthalpy= 0.231380<br>Thermal correction to Gibbs Free Energy= 0.165869<br>Sum of electronic and zero-point Energies= -1142.320344<br>Sum of electronic and thermal Energies= -1142.303822<br>Sum of electronic and thermal Enthalpies= -1142.302878<br>Sum of electronic and thermal Free Energies= -1142.368389<br><br>ν =-611.49
**Name**	**C4-RAF-W**
Cartesian Coordinates	Frequency and Energy
Cl -5.27199100 1.35801300 0.45144300	Zero-point correction= 0.214699 (Hartree/Particle)

N 0.94355400 -0.83949100 -0.19122900<br>N -3.61620300 -0.39960700 -0.55052600<br>N 2.06099100 1.13779500 -0.59706300<br>N 3.78763200 2.84040000 -0.10847300<br>C 0.06424300 -0.59711200 -1.34007100<br>C -1.26233600 -0.04519100 -0.87619400<br>C 2.02399000 -0.00412600 0.08383900<br>C 0.58439700 -1.93255400 0.70589200<br>C -1.37850600 1.24609700 -0.35921800<br>C -2.40911700 -0.82389500 -0.94664700<br>C 2.71785800 -0.17227200 1.40105900<br>C -2.61784000 1.70142100 0.05680800<br>C -3.68714400 0.82155800 -0.06870700<br>C 2.99687000 2.00558900 -0.29776200<br>H -0.08307200 -1.55507000 -1.84112300<br>H 0.56028400 0.09428800 -2.01568300<br>H 0.31349200 -1.53481300 1.68630200<br>H 1.42121400 -2.62225400 0.80962600<br>H -0.26559500 -2.46036600 0.27974700<br>H -0.50749100 1.88775900 -0.28633200<br>H -2.36153000 -1.83273400 -1.34313500<br>H 3.71651300 0.25714800 1.32779900<br>H 2.80118200 -1.21260000 1.70053000<br>H 2.15099900 0.37983400 2.15614000<br>H -2.75656700 2.69554800 0.45952700<br>O 2.95362400 -1.35735700 -0.90636500<br>H 2.78705000 -1.05390700 -1.81379100	Thermal correction to Energy= 0.230717<br>Thermal correction to Enthalpy= 0.231661<br>Thermal correction to Gibbs Free Energy= 0.168472<br>Sum of electronic and zero-point Energies= -1142.315302<br>Sum of electronic and thermal Energies= -1142.299284<br>Sum of electronic and thermal Enthalpies= -1142.298340<br>Sum of electronic and thermal Free Energies= -1142.361529<br><br>ν =-468.34
**Name**	**C5-FHT-W**
Cartesian Coordinates	Frequency and Energy
Cl -5.05200000 2.05863700 0.31041200<br>N 0.91555200 -1.04685700 -0.17577600<br>N -3.53245500 0.62166500 -1.26430400<br>N 1.90700400 0.94600500 -0.70048800<br>N 3.55271900 2.74375500 -0.26873400<br>C -0.06393200 -0.83336500 -1.23438800<br>C -1.28813200 -0.08286900 -0.75550400<br>C 1.85151200 -0.13092000 0.06843400<br>C 0.64128900 -2.13369600 0.77273700<br>C -1.36167300 0.56443600 0.47443500<br>C -2.40541500 -0.02318000 -1.58212200<br>C 2.76630300 -0.33755200 1.22211600<br>C -2.52398300 1.23805800 0.82425000<br>C -3.56331100 1.22368600 -0.09333000<br>C 2.80402400 1.86737100 -0.43111000<br>H -0.36086400 -1.81152800 -1.61452400<br>H 0.40948400 -0.28906000 -2.04988700<br>H -0.30776800 -2.58695600 0.49580000<br>H 0.55982900 -1.75060000 1.79062500<br>H 1.41962500 -2.89665700 0.72866800<br>H -0.52688900 0.55106200 1.16777600<br>H -2.39539700 -0.51850800 -2.54772400<br>H 3.71106400 0.18745000 1.10096500<br>H 2.90852200 -1.37403700 1.51494800<br>H 2.23896500 0.17134400 2.14865800	Zero-point correction= 0.209904 (Hartree/Particle)<br>Thermal correction to Energy= 0.226368<br>Thermal correction to Enthalpy= 0.227313<br>Thermal correction to Gibbs Free Energy= 0.162269<br>Sum of electronic and zero-point Energies= -1142.325299<br>Sum of electronic and thermal Energies= -1142.308835<br>Sum of electronic and thermal Enthalpies= -1142.307891<br>Sum of electronic and thermal Free Energies= -1142.372935<br><br>ν =-1244.26

H -2.62064100 1.75210100 1.77093600<br>O 1.51016500 1.01788000 2.99054100<br>H 1.39404800 1.75923700 2.36712300	
**Name**	**C7-FHT-W**
Cartesian Coordinates	Frequency and Energy
Cl -5.08779200 2.17829900 -0.36630700<br>N 0.87159900 -0.96237800 0.01219700<br>N -3.45259600 0.58151800 -1.64533700<br>N 1.77621300 0.92797800 -0.87134500<br>N 3.40916900 2.78781800 -0.91890700<br>C -0.09038100 -1.00385100 -1.08396200<br>C -1.32029000 -0.16436000 -0.81605100<br>C 1.79022100 0.01248900 0.07628600<br>C 0.70289500 -1.92198900 1.07350000<br>C -1.51361400 0.56977600 0.34982400<br>C -2.32348000 -0.12103300 -1.77899400<br>C 2.75826700 -0.00348300 1.21558900<br>C -2.67991600 1.30608000 0.50798500<br>C -3.59999700 1.26337700 -0.52793000<br>C 2.67001600 1.89270000 -0.84743000<br>H -0.38044200 -2.04632200 -1.22475800<br>H 0.39597400 -0.66793400 -1.99845600<br>H 0.90826400 -1.50250000 2.05543800<br>H 1.41556500 -2.80796600 0.92541700<br>H -0.30343600 -2.33603600 1.03891800<br>H -0.76798900 0.57627500 1.13835200<br>H -2.21382500 -0.67830100 -2.70395100<br>H 3.58368200 0.67865800 1.02321200<br>H 3.14867700 -1.00936500 1.37398200<br>H 2.24624200 0.31268100 2.12892300<br>H -2.86792700 1.88987900 1.39861100<br>O 2.56822000 -3.81317900 0.92021100<br>H 3.10822900 -3.35205700 1.58685000	Zero-point correction= 0.210671 (Hartree/Particle)<br>Thermal correction to Energy= 0.226930<br>Thermal correction to Enthalpy= 0.227875<br>Thermal correction to Gibbs Free Energy= 0.163598<br>Sum of electronic and zero-point Energies= -1142.328095<br>Sum of electronic and thermal Energies= -1142.311835<br>Sum of electronic and thermal Enthalpies= -1142.310891<br>Sum of electronic and thermal Free Energies= -1142.375168<br><br>ν =-1011.13
**Name**	**C8-FHT-W**
Cartesian Coordinates	Frequency and Energy
Cl -4.80086700 2.16102100 0.68226400<br>N 1.03415400 -0.98367500 -0.29570800<br>N -2.55614300 0.84971800 0.96606900<br>N 1.75330500 1.12498600 0.15820800<br>N 3.06373900 2.79672700 1.42860600<br>C 0.16535300 -0.44798500 -1.31697300<br>C -1.07805200 0.23388800 -0.82390600<br>C 1.75553900 -0.15475300 0.47328200<br>C 0.86052900 -2.39704600 0.05552800<br>C -1.93802200 0.83808600 -1.74284800<br>C -1.43443100 0.26995000 0.51772100<br>C 2.51046200 -0.73821700 1.62410100<br>C -3.10006900 1.44157900 -1.29741400<br>C -3.34065800 1.40783400 0.07187300<br>C 2.46977500 1.96787100 0.86885700<br>H -0.08331900 -1.24263000 -2.01952200<br>H 0.75757700 0.32548700 -1.93852600<br>H 0.13696200 -2.50377000 0.86794300<br>H 1.80929400 -2.84005800 0.34857800	Zero-point correction= 0.209059 (Hartree/Particle)<br>Thermal correction to Energy= 0.225961<br>Thermal correction to Enthalpy= 0.226905<br>Thermal correction to Gibbs Free Energy= 0.161453<br>Sum of electronic and zero-point Energies= -1142.328577<br>Sum of electronic and thermal Energies= -1142.311675<br>Sum of electronic and thermal Enthalpies= -1142.310731<br>Sum of electronic and thermal Free Energies= -1142.376183<br><br>ν =-1012.60

H 0.48942900 -2.92281900 -0.82105000<br>H -1.69462000 0.83414900 -2.80058400<br>H -0.80851400 -0.18783200 1.27621600<br>H 2.99023400 0.04613300 2.20500000<br>H 3.27455300 -1.42669100 1.25599100<br>H 1.83131800 -1.30018300 2.26851400<br>H -3.79312300 1.92071300 -1.97541200<br>O 1.27909600 1.12811500 -3.07584600<br>H 0.66210200 0.79902900 -3.75400600	
**Name**	**C9-RAF-W**
Cartesian Coordinates	Frequency and Energy
Cl -4.84867000 1.53035800 1.02516900<br>N 0.95071600 -0.87956400 -0.28094600<br>N -3.34400900 -0.35198100 0.02713900<br>N 1.99016300 1.14006100 -0.51219100<br>N 3.59372100 2.89100400 0.18253500<br>C 0.11564200 -0.59826900 -1.45288600<br>C -1.20117800 0.01894000 -1.04055100<br>C 1.80938100 0.03095400 0.18498900<br>C 0.57252500 -2.04800700 0.51684900<br>C -1.30180700 1.40114400 -0.74805700<br>C -2.25224600 -0.80860500 -0.57861400<br>C 2.52344400 -0.26710500 1.46695700<br>C -2.43082700 1.88214000 -0.12485600<br>C -3.41063300 0.95231900 0.23009100<br>C 2.85674000 2.03374500 -0.09571900<br>H -0.06735000 -1.54189600 -1.96529200<br>H 0.64760300 0.07268200 -2.12086600<br>H -0.13647200 -1.77543300 1.30340900<br>H 1.45016300 -2.50761100 0.96534800<br>H 0.10588800 -2.77436900 -0.14563100<br>H -0.50182800 2.07010400 -1.04116200<br>H -2.20018900 -1.87897800 -0.74879700<br>H 3.14496500 0.57438300 1.76431800<br>H 3.15496700 -1.14961400 1.34475700<br>H 1.80070700 -0.47601600 2.25769000<br>H -2.56137200 2.93328000 0.09322600<br>O -1.89230200 -0.00003700 -2.98085900<br>H -2.71430700 0.50112400 -2.85144200	Zero-point correction= 0.214319 (Hartree/Particle)<br>Thermal correction to Energy= 0.230734<br>Thermal correction to Enthalpy= 0.231678<br>Thermal correction to Gibbs Free Energy= 0.168029<br>Sum of electronic and zero-point Energies= -1142.327860<br>Sum of electronic and thermal Energies= -1142.311445<br>Sum of electronic and thermal Enthalpies= -1142.310501<br>Sum of electronic and thermal Free Energies= -1142.374150<br><br>ν =-369.74
**Name**	**C10-RAF-W**
Cartesian Coordinates	Frequency and Energy
Cl -5.16443400 2.03940900 -0.32849100<br>N 0.81851700 -1.06360400 0.06106000<br>N -3.50791500 0.49275500 -1.62126200<br>N 1.36909400 0.82041100 -1.08889100<br>N 2.73637000 2.84698000 -1.48362400<br>C -0.25424100 -1.28900200 -0.90092600<br>C -1.43589900 -0.37663300 -0.69378900<br>C 1.59806200 0.00702200 -0.06602100<br>C 0.88384700 -1.92186800 1.24951900<br>C -1.67581300 0.31115400 0.49493100<br>C -2.33805400 -0.18469500 -1.76208500<br>C 2.66566800 0.23922700 0.95324300<br>C -2.81029400 1.09054900 0.60437200	Zero-point correction= 0.215151 (Hartree/Particle)<br>Thermal correction to Energy= 0.231143<br>Thermal correction to Enthalpy= 0.232087<br>Thermal correction to Gibbs Free Energy= 0.169907<br>Sum of electronic and zero-point Energies= -1142.329399<br>Sum of electronic and thermal Energies= -1142.313407<br>Sum of electronic and thermal Enthalpies= -1142.312463<br>Sum of electronic and thermal Free Energies= -1142.374643<br><br>ν =-545.71

C -3.68605100 1.11577300 -0.49330600<br>C 2.12951900 1.88260600 -1.25152500<br>H -0.57825400 -2.32620000 -0.80123900<br>H 0.12973300 -1.16481000 -1.91492100<br>H 0.93151100 -1.32057300 2.15705500<br>H 1.74993100 -2.58445100 1.20541500<br>H -0.02097000 -2.52390900 1.28165200<br>H -0.98809100 0.23534100 1.33034000<br>H -2.25629200 -0.80220800 -2.64738900<br>H 3.38849800 0.96735100 0.59084900<br>H 3.17784900 -0.69082200 1.19859400<br>H 2.20401000 0.62574100 1.86685900<br>H -3.03824800 1.64514200 1.50435000<br>O -1.16726400 1.25423400 -2.61552600<br>H -0.32039400 1.12686400 -2.14089200	
**Name**	**C12-RAF-W**
Cartesian Coordinates	Frequency and Energy
Cl -5.34729000 1.41746400 -0.13093000<br>N 0.93216200 -0.99646200 -0.04139300<br>N -3.63239400 -0.04243600 -1.47211200<br>N 1.75128900 0.90824200 -0.98933300<br>N 3.28055000 2.85174200 -1.07481900<br>C -0.04952600 -1.09452500 -1.11250100<br>C -1.32999200 -0.35579600 -0.81384400<br>C 1.76501400 0.04706000 0.01360900<br>C 0.72953100 -1.87099200 1.11719100<br>C -1.47157900 0.55719200 0.21965900<br>C -2.45719300 -0.62155800 -1.62077300<br>C 2.65710400 0.17006500 1.20845300<br>C -2.69197800 1.19737600 0.39050600<br>C -3.71966100 0.91437700 -0.53364400<br>C 2.58761500 1.92124500 -0.98206900<br>H -0.27065200 -2.15050800 -1.27511200<br>H 0.38471000 -0.70472800 -2.03330700<br>H 0.16843000 -1.35794500 1.90246600<br>H 1.68371700 -2.20893000 1.51605500<br>H 0.16506800 -2.74132700 0.78984200<br>H -0.65657700 0.77157200 0.90200400<br>H -2.38359700 -1.37156100 -2.40274300<br>H 3.26312100 1.07088200 1.14580200<br>H 3.31497100 -0.69910600 1.27292700<br>H 2.05525100 0.20564100 2.11918800<br>H -2.85875500 1.91337000 1.18372300<br>O -3.23076000 2.60049600 -1.54431800<br>H -2.33047400 2.39117900 -1.84470000	Zero-point correction= 0.214688 (Hartree/Particle)<br>Thermal correction to Energy= 0.230840<br>Thermal correction to Enthalpy= 0.231785<br>Thermal correction to Gibbs Free Energy= 0.168760<br>Sum of electronic and zero-point Energies= -1142.321348<br>Sum of electronic and thermal Energies= -1142.305195<br>Sum of electronic and thermal Enthalpies= -1142.304251<br>Sum of electronic and thermal Free Energies= -1142.367275<br><br>ν =-535.06
**Name**	**C13-RAF-W**
Cartesian Coordinates	Frequency and Energy
Cl -4.87378300 2.28940700 -0.87455700<br>N 0.86797100 -1.13031900 0.03318800<br>N -3.28270100 0.51132900 -1.93115000<br>N 1.33299500 0.75879300 -1.14061400<br>N 2.44287500 2.94332800 -1.48281300<br>C -0.19345600 -1.41107600 -0.93134900<br>C -1.35789000 -0.45188500 -0.82357800	Zero-point correction= 0.214714 (Hartree/Particle)<br>Thermal correction to Energy= 0.230996<br>Thermal correction to Enthalpy= 0.231940<br>Thermal correction to Gibbs Free Energy= 0.168689<br>Sum of electronic and zero-point Energies= -1142.327603<br>Sum of electronic and thermal Energies= -1142.311322<br>Sum of electronic and thermal Enthalpies= -1142.310378

C 1.55352200 0.01055100 -0.07238700<br>C 0.92817900 -1.94851500 1.24701300<br>C -1.61260800 0.30744200 0.30322500<br>C -2.21604300 -0.30437400 -1.91428900<br>C 2.51604000 0.36692000 1.01572800<br>C -2.66018900 1.24870800 0.28212400<br>C -3.49226700 1.23274500 -0.86014800<br>C 1.95517400 1.90768800 -1.27107800<br>H -0.53284700 -2.43195600 -0.75826600<br>H 0.21183500 -1.36335800 -1.94258000<br>H 0.34848400 -1.49616900 2.05610300<br>H 1.95840200 -2.07892300 1.57098900<br>H 0.51421700 -2.92737400 1.01829600<br>H -0.98670600 0.24478300 1.18662900<br>H -2.04265200 -0.87542900 -2.82037300<br>H 2.96351900 1.34076700 0.83003500<br>H 3.30785000 -0.38325200 1.07225200<br>H 1.99983000 0.38560600 1.97798700<br>H -2.98868000 1.73968800 1.18654500<br>O -1.57381200 2.90173100 -0.21441700<br>H -0.96879400 2.47914700 -0.84753400	Sum of electronic and thermal Free Energies= -1142.373628<br><br>ν =-441.99
**Name**	**C14-RAF-W**
Cartesian Coordinates	Frequency and Energy
Cl -5.38604900 1.25293800 0.26951800<br>N 0.92831900 -0.80926000 -0.17229300<br>N -3.70513800 -0.38473200 -0.88822000<br>N 2.19870400 0.94583600 -0.90377400<br>N 4.06674800 2.55269700 -0.68602100<br>C 0.02941200 -0.69106900 -1.32158800<br>C -1.31212100 -0.15531100 -0.90468700<br>C 1.92833000 0.04988600 0.03201700<br>C 0.49617800 -1.74761700 0.86631100<br>C -1.41837400 1.07584400 -0.20424100<br>C -2.47977900 -0.81960500 -1.22749700<br>C 2.69740700 -0.06752500 1.31174200<br>C -2.69706100 1.48975100 0.22330300<br>C -3.77805200 0.72989600 -0.18689900<br>C 3.20258400 1.77378700 -0.73454300<br>H -0.09084900 -1.67874300 -1.76892700<br>H 0.48382100 -0.02808900 -2.05560800<br>H -0.07425300 -1.23520200 1.64517600<br>H 1.35007300 -2.25268800 1.31237200<br>H -0.14023600 -2.49599900 0.39687400<br>H -0.54099700 1.51763800 0.25371500<br>H -2.44075000 -1.75232700 -1.77929600<br>H 3.41663900 0.74301300 1.40353100<br>H 3.23214800 -1.01990000 1.33444100<br>H 2.01676800 -0.04209800 2.16438600<br>H -2.82536000 2.38530000 0.81566900<br>O -1.20070900 2.31298700 -1.74026500<br>H -1.98947700 2.07561100 -2.25801700	Zero-point correction= 0.214014 (Hartree/Particle)<br>Thermal correction to Energy= 0.230510<br>Thermal correction to Enthalpy= 0.231454<br>Thermal correction to Gibbs Free Energy= 0.167410<br>Sum of electronic and zero-point Energies= -1142.323093<br>Sum of electronic and thermal Energies= -1142.306597<br>Sum of electronic and thermal Enthalpies= -1142.305652<br>Sum of electronic and thermal Free Energies= -1142.369697<br><br>ν =-553.24

References

1. M. G. Evans and M. Polanyi, *Trans. Faraday Soc.*, 1935, **31**, 875-894.
2. H. Eyring, *J. Chem. Phys.*, 1935, **3**, 107-115.
3. D. G. Truhlar, W. L. Hase and J. T. Hynes, *J. Phys. Chem.*, 1983, **87**, 2664-2682.
4. T. Furuncuoglu, I. Ugur, I. Degirmenci and V. Aviyente, *Macromolecules*, 2010, **43**, 1823-1835.
5. E. Vélez, J. Quijano, R. Notario, E. Pabón, J. Murillo, J. Leal, E. Zapata and G. Alarcón, *J. Phys. Org. Chem.*, 2009, **22**, 971-977.
6. E. Pollak and P. Pechukas, *J. Am. Chem. Soc.*, 1978, **100**, 2984-2991.
7. A. Fernández-Ramos, B. A. Ellingson, R. Meana-Pañeda, J. M. Marques and D. G. Truhlar, *Theor. Chem. Acc.*, 2007, **118**, 813-826.
8. C. Eckart, *Phy. Rev.*, 1930, **35**, 1303.
9. R. A. Marcus, *Annu. Rev. Phys. Chem.*, 1964, **15**, 155-196.
10. R. A. Marcus, *Rev. Mod. Phys.*, 1993, **65**, 599.
11. Y. Lu, A. Wang, P. Shi and H. Zhang, *PloS one*, 2017, **12**, e0169773.
12. Y. Lu, A. Wang, P. Shi, H. Zhang and Z. Li, *PloS one*, 2015, **10**, e0133259.
13. S. F. Nelsen, S. C. Blackstock and Y. Kim, *J. Am. Chem. Soc.*, 1987, **109**, 677-682.
14. S. F. Nelsen, M. N. Weaver, Y. Luo, J. R. Pladziewicz, L. K. Ausman, T. L. Jentzsch and J. J. O'Konek, *J. Phys. Chem. A*, 2006, **110**, 11665-11676.
15. A. Galano and J. R. Alvarez-Idaboy, *J. Comput. Chem.*, 2013, **34**, 2430-2445.
16. F. C. Collins and G. E. Kimball, *J. Colloid Sci.* , 1949, **4**, 425-437.
17. M. Von Smoluchowski, *Z. Phys. Chem*, 1917, **92**, 129-168.
18. D. G. Truhlar, *J. Chem. Educ.*, 1985, **62**, 104.
19. A. Einstein, *Ann. Phys.*, 1905, **17**, 549-560.
20. G. G. Stokes, *Mathematical and Physical Papers*, University Press, Cambridge, 1905.
21. A. Galano and J. Raúl Alvarez-Idaboy, *Int. J. Quantum Chem.*, 2019, **119**, e25665.
22. Q. V. Vo, T. V. Gon, M. V. Bay and A. Mechler, *J. Phys. Chem. B*, 2019, **123**, 10672-10679.
23. Q. V. Vo, N. M. Tam, M. Van Bay, N. M. Thong, T. Le Huyen, N. T. Hoa and A. Mechler, *RSC Adv.*, 2020, **10**, 14937-14943.
